# Lef1 restricts ectopic crypt formation and tumor cell growth in intestinal adenomas

**DOI:** 10.1126/sciadv.abj0512

**Published:** 2021-11-17

**Authors:** Sarika Heino, Shentong Fang, Marianne Lähde, Jenny Högström, Sina Nassiri, Andrew Campbell, Dustin Flanagan, Alexander Raven, Michael Hodder, Nadia Nasreddin, Hai-Hui Xue, Mauro Delorenzi, Simon Leedham, Tatiana V. Petrova, Owen Sansom, Kari Alitalo

**Affiliations:** 1 Translational Cancer Medicine Program (CAN-PRO), iCAN Digital Precision Cancer Medicine Flagship and Wihuri Research Institute, Faculty of Medicine, HiLIFE-Helsinki Institute of Life Science, Biomedicum Helsinki, University of Helsinki, 00014 Helsinki, Finland.; 2 School of Biopharmacy, China Pharmaceutical University, Nanjing 211198, P.R. China.; 3 Bioinformatics Core Facility, SIB Swiss Institute of Bioinformatics, Lausanne, Switzerland.; 4 Cancer Research UK Beatson Institute, Garscube Estate, Glasgow G61 1BD, UK.; 5 Institute of Cancer Sciences, Garscube Estate, Glasgow G61 1QH, UK.; 6 Intestinal Stem Cell Biology Laboratory, Wellcome Trust Centre for Human Genetics, University of Oxford, Oxford OX3 7BN, UK.; 7 Center for Discovery and Innovation, Hackensack University Medical Center, Nutley, NJ 07110, USA.; 8 Department of Oncology, University of Lausanne and CHUV, Epalinges, Switzerland.; 9 Ludwig Institute for Cancer Research Lausanne, Epalinges, Switzerland.

## Abstract

Somatic mutations in *APC* or *CTNNB1* genes lead to aberrant Wnt signaling and colorectal cancer (CRC) initiation and progression via-catenin–T cell factor/lymphoid enhancer binding factor TCF/LEF transcription factors. We found that *Lef1* was expressed exclusively in *Apc*-mutant, Wnt ligand–independent tumors, but not in ligand-dependent, serrated tumors. To analyze *Lef1* function in tumor development, we conditionally deleted *Lef1* in intestinal stem cells of *Apc^fl/fl^* mice or broadly from the entire intestinal epithelium of *Apc^fl/fl^* or *Apc^Min/+^* mice. Loss of *Lef1* markedly increased tumor initiation and tumor cell proliferation, reduced the expression of several Wnt antagonists, and increased *Myc* proto-oncogene expression and formation of ectopic crypts in *Apc*-mutant adenomas. Our results uncover a previously unknown negative feedback mechanism in CRC, in which ectopic *Lef1* expression suppresses intestinal tumorigenesis by restricting adenoma cell dedifferentiation to a crypt-progenitor phenotype and by reducing the formation of cancer stem cell niches.

## Teaser

Lef1 is induced in intestinal adenomas, in which it restricts ectopic crypt formation and tumor growth.

## INTRODUCTION

Aberrant activation of Wnt signaling is a hallmark of colorectal cancer (CRC) (*1*). Intestinal stem cells (ISCs), which self-renew and generate multipotent progenitor cells in the gut, are central in CRC initiation and progression (*2*, *3*). The rapidly proliferating ISCs, marked by the leucine-rich repeat-containing G protein–coupled receptor 5 (Lgr5), reside at the bottom of intestinal crypts, interspersed between Paneth cells (*4*), which provide essential niche signals to promote Lgr5^+^ stem cell renewal (*5*).

The canonical Wnt signaling pathway is a critical regulator of ISCs, and aberrant activation of the Wnt pathway, most commonly via loss of function of the *Adenomatous polyposis coli* (*APC*) gene, is a key initiating step in their malignant transformation (*1*, *2*, *6*). Activation of this pathway leads to the formation and nuclear translocation of β-catenin–T cell factor/lymphoid enhancer binding factor 1 (Tcf/Lef1) complexes that activate transcription of oncogenic target genes, such as the *Prospero homeobox 1* (*Prox1*) and *Myc* (*7*–*10*). Furthermore, non-ISCs can also initiate intestinal tumorigenesis after acquisition of additional genetic alterations, such as oncogenic mutations in the *Kirsten rat sarcoma viral oncogene homolog* (*KRAS*) (*1*, *11*–*13*). This leads to dedifferentiation of *Apc*-mutant cells and to the formation of crypts in intestinal villi (ectopic crypts) via activation of KRAS downstream effectors, such as mammalian target of rapamycin (mTOR), mitogen-activated protein kinase kinase 1 (MEK-1) and MEK-2, extracellular signal–regulated kinase, phosphoinositide 3-Kinase (PI3K), or Akt (*1*, *13*, *14*). Furthermore, decreased transforming growth factor–β1 (TGFβ) signaling enhances the ability of *Kras^G12D/+^* mutation to drive dedifferentiation and markedly accelerates tumorigenesis in the *Apc*-mutant intestine (*14*).

*APC* and *CTNNB1* mutations lead to constitutive activation of the Wnt pathway, in which the tumors progress in a Wnt ligand–independent manner (*15*). CRC can also develop through an alternative serrated trajectory that involves distinct genetic alterations, such as truncating *Ring finger protein 43* (*RNF43*) mutations or *R-spondin* (*RSPO*) fusions (*16*, *17*). These tumors remain ligand dependent and show increased sensitivity to Wnt ligands from adjacent cells in the tumor microenvironment.

The TCF/LEF1 family comprises four members TCF1, LEF1, TCF3, and TCF4, encoded by the *TCF7*, *LEF1*, *TCF7L1*, and *TCF7L2* genes, respectively. The complexity of the TCF/LEF1 transcriptional network is further exacerbated by alternative splicing, which can generate β-catenin binding and antagonistic isoforms (*18*, *19*). In the adult intestine, *Tcf4* is critical for crypt homeostasis, whereas *Tcf1* and *Tcf3* are dispensable (*20*, *21*). Intestinal adenomas maintain high levels of *Tcf4* transcripts and low *Tcf3* levels (*9*). Although Tcf1 expression is strongly increased during tumorigenesis, approximately 15% of *Tcf7-*deficient mice develop intestinal neoplasias by 12 months of age and in *Apc*-mutant mice, *Tcf7* deletion markedly increases tumorigenesis, indicating that *Tcf1* is a tumor suppressor in the intestine (*9*, *22*). *Lef1* is the only member of the *Tcf* gene family that is not expressed in the normal intestine but is induced during intestinal tumorigenesis (*9*, *23*). The function of *Lef1* has been analyzed mainly in the context of embryonic development and lymphocyte differentiation (*24*, *25*). Aberrant expression of *LEF1* has been reported in human leukemia, lymphoma, lung adenocarcinoma, prostate cancer, and CRC (*23*, *26*–*28*). A previous report showed that short hairpin–RNA–mediated silencing of *LEF1* decreased the growth of subcutaneous human CRC xenografts (*29*). Here, we have assessed the role of *Lef1* using multiple genetic models of intestinal adenomas and CRC progression. We show that *Lef1* is expressed in Wnt ligand–independent *Apc*-mutant adenomas, but not in Wnt ligand–dependent *Rnf43;Znrf3-*mutant intestine. Our results lead us to conclude that the expression of *Lef1* in *Apc*-deficient epithelial cells blunts tumor initiation and growth by restricting MYC activity and the number of tumor-associated ectopic crypts that provide niches for tumor stem cells.

## RESULTS

### LEF1 is expressed in Wnt ligand–independent but not in Wnt ligand–dependent CRCs

**Fig. 1.**
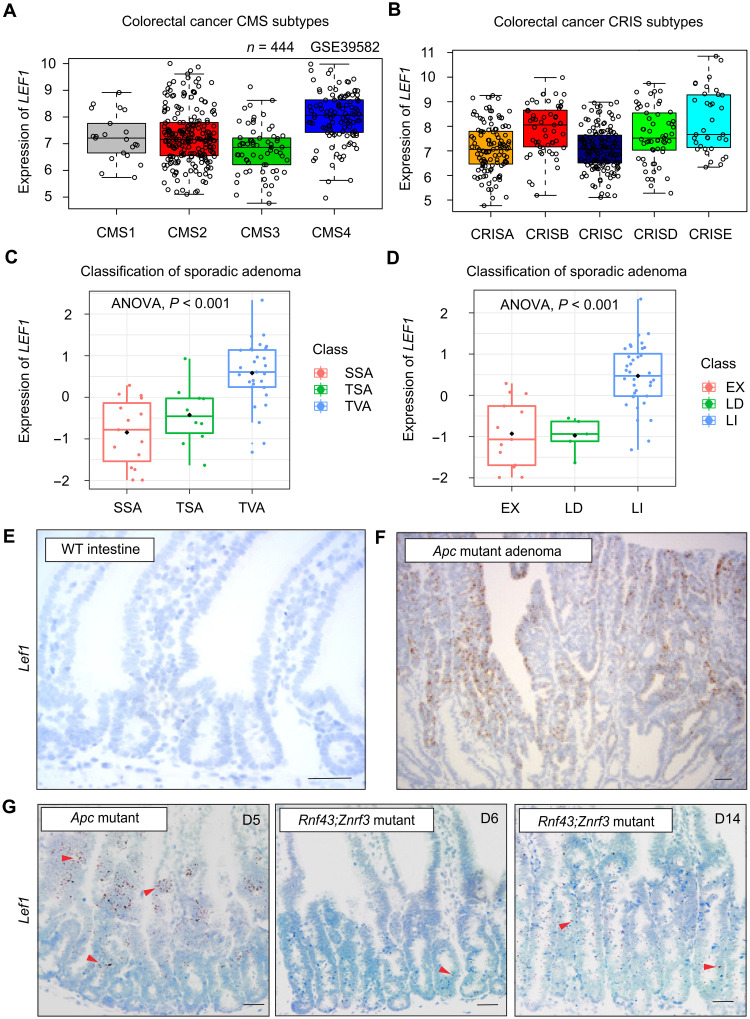
LEF1 is expressed in Wnt ligand–independent but not in Wnt ligand–dependent CRCs. (**A** and **B**) Analysis of LEF1 expression in the CRC (A) CMS and (B) CRIS subtypes. Data obtained from GSE39582. (**C**) Analysis of LEF1 expression (*z* score) in the sessile serrated adenoma (SSA), traditional serrated adenoma (TSA), and tubulovillous adenoma (TVA) CRC subtypes. (**D**) Analysis of LEF1 expression (*z* score) in the CRC subtypes. LI (ligand-independent tumor) has β-catenin mutation or *APC* mutation; LD (ligand-dependent tumor) has RSPO3 fusion or RNF43 mutation; and EX indicates samples without a known WNT alteration. (**E** and **F**) In situ hybridization of *Lef1* (brown signal) in (E) adjacent normal tissue and (F) *Apc-*mutant adenoma. (**G**) In situ hybridization of *Lef1* (brown signal) in Apc^fl/fl^;Villin-Cre^ERT2^ intestine 5 days after tamoxifen and in Rnf43^fl/fl^;Znrf3^fl/fl^;Villin-Cre^ERT2^ intestine 6 and 14 days after tamoxifen. Arrowheads point *Lef1*^+^ cells. Scale bars, 50 μm.

Analysis of *LEF1* in CRC (*n* = 444) consensus molecular subtypes (CMSs) showed its highest expression in primary tumors of the CMS4 subtype, which show abundant stromal infiltration and a dismal prognosis (Fig. 1A) (*30*). The lowest *LEF1* expression levels were found in the CMS3 subtype (Fig. 1A), which is characterized by *KRAS* activation and metabolic deregulation (*30*). Consistent with this, analysis of CRC intrinsic subtypes (CRISs) showed the lowest expression of *LEF1* in CRISA and CRISC subtypes (Fig. 1B), characterized by *KRAS* mutations and increased *MYC* transcripts, respectively (*31*).

We next analyzed *LEF1* expression in conventional and serrated CRCs. Conventional tubular or tubulovillous adenomas (TVAs) are ligand independent, and their tumor-initiating *APC* or *CTNNB1* mutations likely occur in stem cells (*2*). The origin of sessile serrated adenomas (SSAs) and traditional serrated adenomas (TSAs) is not known, but they have been suggested to be derived from ectopic crypt foci (*32*). The Wnt ligand–dependent serrated tumors, which form approximately 35% of sporadic CRCs, are typically associated with *BRAF* or *KRAS* mutations, microsatellite instability, and a poor prognosis (*16*, *17*, *33*). *LEF1* was expressed in TVAs but not in SSAs or TSAs (Fig. 1C). Consistently with this, ligand-dependent tumors and tumors without a known Wnt alteration expressed little or no *LEF1* compared to the *LEF1* positive Wnt ligand–independent tumors (Fig. 1D).

We analyzed *Lef1* expression in *Apc*-mutant tumors that are ligand independent and in *Rnf43;Znrf3*-mutant adenomas that are ligand dependent and have a serrated growth pattern. We first confirmed that in situ hybridization detects *Lef1* expression in the *Apc* adenomas but not in adjacent normal tissue (Fig. 1, E and F). *Lef1* was also expressed in the intestinal cells of the *Villin-Cre^ERT2^;Apc^fl/fl^* mice, but not in the *Villin-Cre^ERT2^; Rnf43^fl/fl^; Znrf3^fl/fl^* mice, in which 14 days after *Rnf43* and *Znrf3* deletion, only a low level of *Lef1* expression was detected (Fig. 1G). Collectively, these results indicate that Lef1 is activated only in the Wnt ligand–independent *Apc*-mutant tumors, but not in the ligand-dependent *Rnf43* and *Znrf3* mutant tumors.

### Lef1 is not required for homeostasis or regeneration of the healthy intestine

To confirm that *Lef1* is not required for homeostasis in the healthy intestine, we deleted *Lef1* from Lgr5^+^ ISCs of 8-week-old *Lgr5-EGFP-IRES-Cre^ERT2^;Lef1^fl/fl^* (*Lef1*^Δ*/*Δ^) mice and compared their intestines with the intestines of *Lgr5-EGFP-IRES-Cre^ERT2^* (WT) control mice 1 week after the deletion (fig. S1A). We found no differences in intestinal histology or upon immunohistochemical analysis of cells positive for Prox1, E-cadherin, chromogranin A (ChgA; a marker for neuroendocrine cells), lysozyme 1 (Lyz1; Paneth cells), mucin 2 (Muc2; goblet cells), doublecortin-like kinase 1 (tuft cells), Lgr5–green fluorescent protein (GFP)^+^ stem cells, or 5-ethynyl-2′-deoxyuridine (EdU)^+^ proliferating cells (fig. S1B). Moreover, Lef1 was not expressed in the intestines harvested during epithelial repair at 3, 24, and 72 hours after 10-gray (Gy) irradiation, showing that Lef1 expression is not activated upon regeneration of the intestinal epithelium (fig. S1C).

### Lef1 is induced after *Apc* gene deletion in stem cells

Next, we investigated the onset of *Lef1* expression in the development of intestinal ligand–independent tumors. Tamoxifen was used to delete *Apc* in Lgr5^+^ cells of *Lgr5-EGFP-IRES-Cre^ERT2^;Apc^fl/fl^* (*LApc*) mice. The mice were euthanized at various time points, and sections from the gut were stained for Lef1 and for the Wnt target Prox1, which has been shown to promote tumor progression (fig. S2, A and B) (*7*). Consistent with previous findings (*7*, *34*), Prox1 expression was activated 4 days after *Apc* deletion. Lef1 expression was induced 10 days after *Apc* inactivation almost exclusively in the Prox1^+^ adenoma cells (96.01 ± 10.78%) (fig. S2B). Furthermore, most of the cells expressing the β-catenin–binding Lef1 isoform (69.89 ± 28.22%) were GFP-Lgr5^+^ cells, indicating that Lef1 was induced in the transformed intestinal progenitor cells (fig. S2B). Analysis of cell proliferation by EdU labeling revealed that the Lef1^+^ adenoma cells proliferate less than the Lef1^−^ adenoma cells (fig. S2C). These results show that after Wnt signaling activation and the consequent onset of adenoma development, Lef1 expression occurs in slowly proliferating cancer stem cells (CSCs).

### *Lef1* deletion increases the initiation and growth of *Apc*-mutant adenomas

**Fig. 2.**
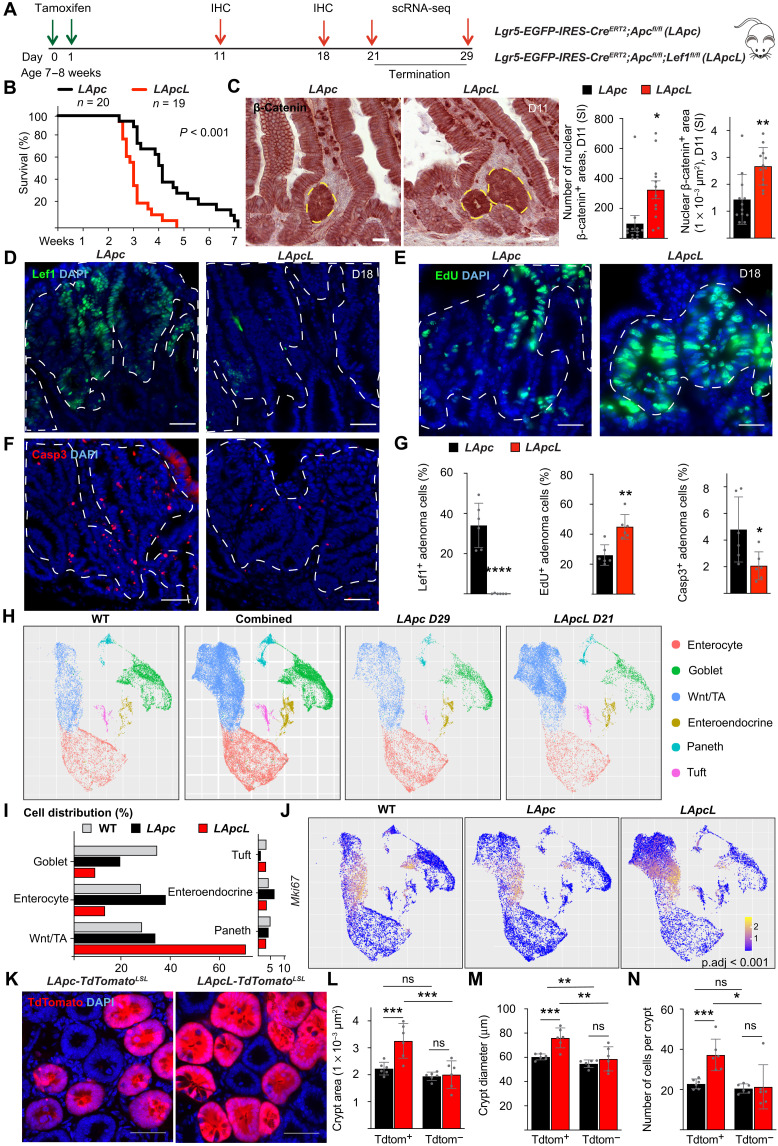
*Lef1* deletion increases tumor initiation and growth in *Apc*-mutant adenomas. (**A**) Schematic of the experiment. IHC, immunohistochemistry. (**B**) Kaplan-Meier survival curves of the *LApc* (*n* = 20) and *LApcL* (*n* = 19) mice. (**C**) Staining and quantification (means ± SEM) of nuclear β-catenin^+^ area in the small intestine (SI) 11 days after gene deletion. Scale bars, 100 μm. *n* = 12 per group, *P < 0.05 and **P < 0.01. (**D** to **G**) Immunostaining and quantification (means ± SD) of the percentage of (D and G) Lef1^+^, (E and G) EdU^+^, and (F and G) Casp3^+^ adenoma cells 18 days after gene deletion. Scale bars, 50 μm. *n* = 6 per group, *P < 0.05, **P < 0.01, and ****P < 0.001. (**H**) UMAP visualization of scRNA-seq results from epithelial cellular adhesion molecule (EpCAM)^+^ intestinal cells of the WT, *LApc*, and *LApcL* mice at days 29 and 21, respectively. (**I**) Cell distribution percentages in the indicated scRNA-seq clusters of WT, *LApc*, and *LApcL* cells. (**J**) *Mki67* RNA expression in WT, *LApc*, and *LApcL* epithelial cells. Adjusted *P* value (p.adj) indicates the significance of the *Mki67* expression in the *LApcL* versus *LApc* adenoma cells. (**K**) Representative images of *LApc-TdTomato^LSL^* and *LApcL-Tdtomato^LSL^* crypts. Scale bars, 50 μm. (**L** to **N**) Quantification of the (L) crypt area, (M) crypt diameter, and (N) crypt cell number of *LApc-Tdtomato^LSL^* and *LApcL-TdTomato^LSL^* intestines. *n* = 6 per group, *P < 0.05, **P < 0.01, and ***P < 0.005. The dashed lines in (C) to (F) indicate the nuclear β-catenin^+^ adenoma cell area. ns, not significant.

To define Lef1 function in stem cells and in ligand-independent intestinal tumorigenesis, we deleted *Apc* with and without *Lef1* in the Lgr5^+^ stem cells of *Lgr5-EGFP-IRES-Cre^ERT2^*;*Apc^fl/fl^;Lef1^fl/fl^* (*LApcL*) or *LApc* mice (Fig. 2A). Unexpectedly, we noticed a significant decrease in the survival of the mice after *Lef1* deletion (Fig. 2B). Analysis of β-catenin in tissue sections from the gut showed that the *LApcL* mice had larger and more numerous adenomas than the control *LApc* mice (Fig. 2C). Quantification of EdU^+^ nuclei showed a higher proportion of proliferating adenoma cells in *LApcL* tumors than in *LApc* tumors (Fig. 2, D, E, and G), whereas immunostaining of cleaved caspase 3 (Casp3) was decreased (Fig. 2, F and G). Thus, both increased proliferation and decreased apoptosis contribute to faster tumor growth in mice with combined deletion of *Apc* and *Lef1*.

To further analyze the effects of *Lef1* deletion, we performed single-cell RNA sequencing (scRNA-seq) analysis of sorted epithelial cell adhesion molecule (EpCAM)^+^ intestinal epithelial cells from wild-type (WT), *LApc*, and *LApcL* mice at time points when they met the criteria for euthanasia (days 29 and 21, respectively). A previous scRNA-seq study of 53,193 normal EpCAM^+^ intestinal epithelial cells defined transcriptional signatures for each of the main types of differentiated cells in the small intestine (*35*). Unsupervised clustering partitioned our cells into six distinct cell types of which enterocytes, goblet, Paneth, enteroendocrine, and tuft cells are reported in the WT intestine (Fig. 2H and fig. S3A) (*35*). The remaining cluster, which we termed Wnt/TA, contained both normal and transformed transit-amplifying (TA) cells that expressed several known markers of the Wnt signaling pathway and cell proliferation, such as *Lgr5*, *Prox1*, *Notum*, and *Ptma* (Fig. 2H and fig. S3A).

We found that the Wnt/TA cell population was increased after *Apc* deletion and a further increase occurred after deletion of both *Apc* and *Lef1* (Fig. 2, H and I and fig. S3A). The Wnt/TA cell population comprised 70% of all EpCAM^+^ cells in the *LApcL* intestine but only 33% in the *LApc* intestine and 28% in the adjacent normal tissue, indicating that *Lef1* deletion led to an increase in adenoma cells (Fig. 2I). Accordingly, the proportion of enterocytes and goblet cells was strongly decreased in the *LApcL* versus *LApc* mice (33.4% versus 44.3%, respectively) (Fig. 2, H and I and fig. S3A). To further analyze the adenoma cells, we subclustered the Wnt/TA cell population and excluded the nonmalignant cell clusters that were common to be WT, *LApc*, and *LApcL* intestines. This analysis indicated that the *LApcL* mice had 4.6-fold more adenoma cells within the Wnt/TA cluster than the *LApc* mice (fig. S3B). Moreover, in agreement with increased EdU incorporation, *Lef1* deletion increased the expression of the cell cycle marker *Mki67* in the adenoma cells (Fig. 2J and fig. S3C).

To better distinguish adenoma cells from the normal epithelium, we crossed the *LApcL* mice with *tdTomato^LSL^* reporter mice and compared their crypts to the crypts in *LApc-tdTomato^LSL^* mice 21 days after gene deletion. The TdTomato^+^ crypt bottoms were enlarged in the *LApcL* mice and had larger diameters and more cells than the corresponding *LApc* crypts (Fig. 2, K to N), suggesting that *Lef1* deletion increases the number of aberrant crypt foci, previously identified as precursors of CRC (*36*). Overall, the immunostaining and scRNA-seq data demonstrate that *Lef1* inactivation increases the proportion of *Apc-*mutant adenoma cells that have a high Wnt signaling activity.

### *Lef1* deletion decreases expression of Wnt antagonists but increases Myc and Cd44

**Fig. 3.**
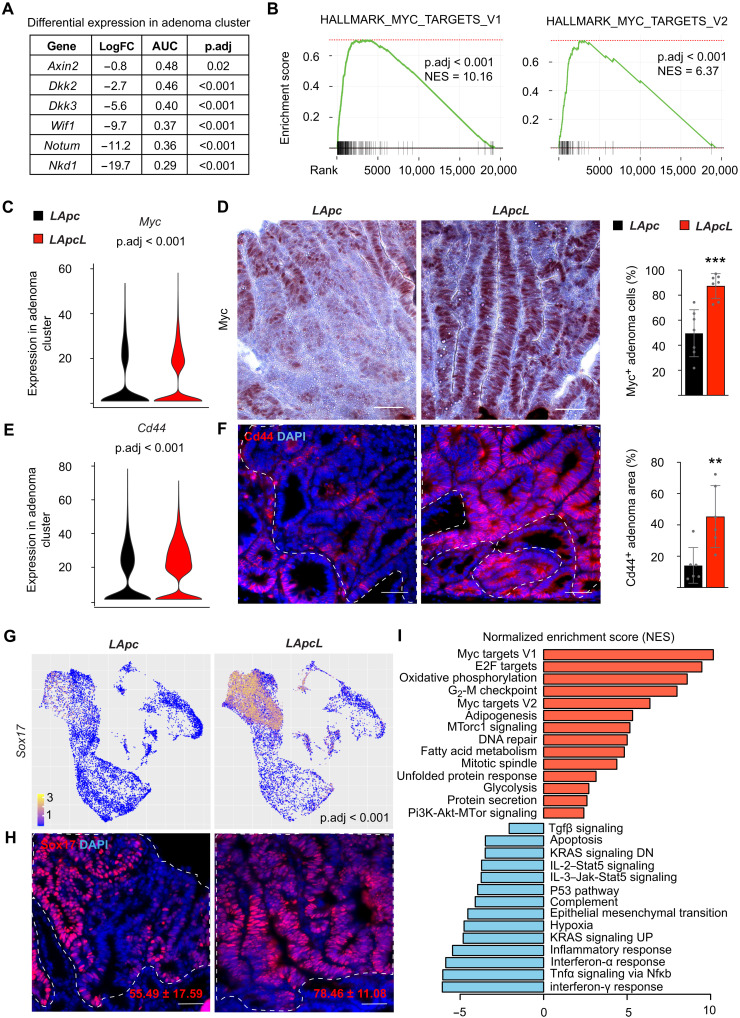
*Lef1* deletion decreases expression of Wnt antagonists but increases Myc and Cd44. (**A**) Differential gene expression analysis of Wnt antagonists *Nkd1*, *Notum*, *Wif1*, *Dkk3*, *Dkk2*, and *Axin2* in *LApcL* versus *LApc* adenoma cluster. LogFC, log fold change. (**B**) GSEA of MSigDB’s Myc signaling Hallmark gene sets in the *LApcL* versus *LApc* adenoma cluster. NES, normalized enrichment score. (**C**) *Myc* expression based on scRNA-seq analysis of the *LApc* and *LApcL* adenoma cluster. (**D**) Myc immunostaining and quantification of Myc^+^ cells in *LApc* and *LApcL* tumor sections 18 days after gene deletion. Scale bars, 50 μm. *n* = 6 per group, ***P < 0.005. (**E**) *Cd44* expression based on scRNA-seq analysis of the *LApc* and *LApcL* adenoma cluster. (**F**) Cd44 immunostaining and quantification in *LApc* and *LApcL* adenoma cells 18 days after gene deletion. Scale bars. 50 μm; *n* = 6 per group, **P < 0.01. (**G**) *Sox17* expression based on scRNA-seq analysis of the *LApc* and *LApcL* adenoma clusters. (**H**) Sox17 immunostaining and quantification in *LApc* and *LApcL* adenoma cells 18 days after gene deletion. Scale bars, 50 μm. *n* = 6 per group. (**I**) GSEA analysis showing selected MSigDB’s Hallmark pathways, which are significantly (*P* < 0.05) enriched in the *LApcL* versus *LApc* adenoma cluster. The dashed lines indicate nuclear β-catenin^+^ adenoma areas. Tnfa, tumor necrosis factor–α; Nfκb, nuclear factor κB; Jak, Janus kinase; Stat5, signal transducers and activators of transcription 5.

Gene set enrichment analysis (GSEA) of the scRNA-seq data from the adenoma cells showed that *Lef1* deletion increased the overall strength of the Wnt signaling and both *Tcf7* and *Tcf7l2* transcripts were increased significantly after *Lef1* deletion in the adenoma cells (fig. S4, A and D, and table S1). Differential gene expression analysis of the scRNA-seq data from the adenoma cells showed substantially fewer transcripts encoding the Wnt antagonists *Axin2*, *Dkk2*, *Dkk3*, *Wif1*, *Notum*, and *Nkd1* in the *LApcL* mice than in the *LApc* mice (Fig. 3A and table S1). Furthermore, comparison of the transcriptional signatures of intestinal epithelial cells from the *LApcL* versus *LApc* and from the *VApc^fl/+^* versus *WT* mice showed that the 250 most down-regulated genes in the *Lef1* signature were up-regulated in the *Apc* signature (fig. S4B). Previous studies have shown that enhanced expression of the Wnt target gene *Myc* is required for the growth of mouse adenomas and human CRCs and that deletion of the *Myc-335* regulatory element makes mice resistant to intestinal tumorigenesis (*10*, *37*). The GSEA analysis of hallmark pathways showed that *Myc* and its target transcripts in the adenoma cluster were more up-regulated after *Apc;Lef1* deletion than after *Apc* deletion (Fig. 3, B, C, and I), and immunostaining confirmed a corresponding increase in Myc protein levels (Fig. 3D).

scRNA-seq analysis indicated that *Lef1* deletion increased transcripts encoding *Cd44*, which is a Wnt target, a CSC marker, and a key driver of intestinal tumorigenesis (Fig. 3E) (*38*–*40*). The increase in Cd44 protein in *LApcL* adenomas was confirmed by immunostaining (Fig. 3F). scRNA-seq analysis indicated that *Lef1* deletion increases also the expression of *Sox17*, which is a direct target of Myc; a consistent increase in the Sox17 protein was observed in immunofluorescence analysis (Fig. 3, G and H) (*10*). Consistent with increased biosynthesis and cell growth driven by Myc (*41*, *42*), we observed a notable increase in ribosomal gene expression in the *LApcL* tumor cells (fig. S4C and table S1). Gene ontology enrichment analysis further confirmed highly increased RNA metabolism and ribosomal biogenesis upon *Lef1* deletion (table S2). Furthermore, a comparison of *LApc* and *LApcL* adenoma cells in GSEA pathway analysis showed a significant increase in mTorc1 and Pi3K-Akt/mTor signaling and a decrease in Tgfβ signaling after *Lef1* deletion (Fig. 3I). Collectively, these results suggest that increased Myc and Cd44 signaling underlie enhanced tumorigenesis in the *LApcL* intestine.

### *Lef1* deletion in *Apc*-mutant adenomas decreases Lgr5^+^ stem cells but increases primary organoid formation

**Fig. 4.**
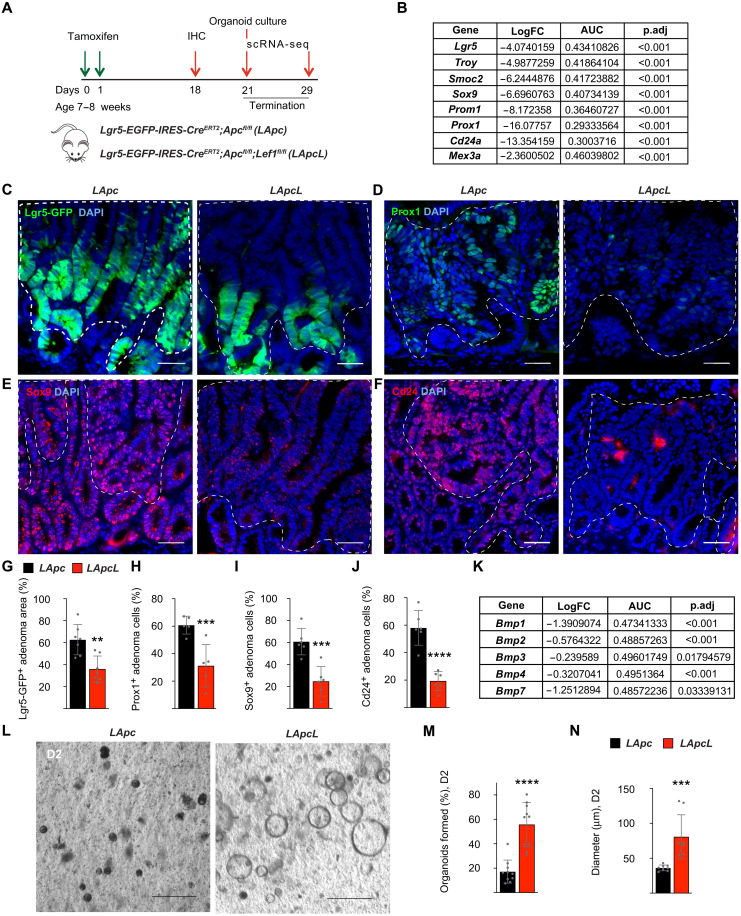
*Lef1* deletion in *Apc*-mutant adenomas decreases Lgr5^+^ stem cells but increases primary organoid formation. (**A**) Schematic of the experiment. (**B**) Differential gene expression analysis of the stem cell marker transcripts *Lgr5*, *Troy*, *Smoc2*, *Sox9*, *Prom1*, *Prox1*, *Cd24a*, and *Mex3a* in *LApcL* versus *LApc* adenoma cluster. (**C** to **J**) Immunostaining and quantification of (C and G) Lgr5-GFP, (D and H) Prox1, (E and I) Sox9, and (F and J) Cd24 in *LApc* and *LApcL* adenomas. The dashed lines indicate nuclear β-catenin^+^ adenoma areas. Scale bars, 50 μm. *n* = 5 to 7 per group, **P < 0.01, ***P < 0.005, and ****P < 0.001. (**K**) Differential gene expression analysis of stem cell markers *Bmp1*, *Bmp2*, *Bmp3*, *Bmp4*, and *Bmp7* in *LApcL* versus *LApc* adenoma cluster. (**L**) Representative images of *LApc* and *LApcL* organoids and quantification of the organoid (**M**) formation and (**N**) diameter 2 days after crypt isolation and Matrigel embedding. Scale bars, 200 μm. *n* = 8 to 10 wells of organoids were counted; ***P < 0.005 and ****P < 0.001.

Given that ISCs fuel CRC progression (*2*, *3*), we asked how *Lef1* deletion affects the CSCs. Unexpectedly, despite the increased expression of Cd44, we observed an overall reduction in transcripts encoding the stem cell markers *Lgr5*, *Troy*, *Smoc2*, *Sox9*, *Prom1*, *Prox1*, *Cd24a*, and *Mex3a* in the *LApcL* mice (Fig. 4, A and B) (*2*, *3*, *34*, *38*, *43*–*47*). Immunofluorescence analysis confirmed a corresponding decrease in the Lgr5-GFP^+^ tumor cell area and Sox9^+^, Prox1^+^, and Cd24^+^ adenoma cells in the intestinal sections 18 days after *Lef1* deletion (Fig. 4, C to J). Despite the decrease in CSC numbers, we also found a decrease in transcripts encoding bone morphogenetic protein 1 (Bmp1) to Bmp4 and Bmp7, which inhibit stem cell expansion in the normal intestine (Fig. 4K) (*48*).

To study the ability of the *Apc;Lef1*-deleted tumor cells to form organoids in culture, we isolated intestinal cells from *LApc* and *LApcL* mice 21 days after tamoxifen treatment and cultured them in growth factor–deficient Matrigel. We found that, when first plated into Matrigel, the freshly isolated *LApcL* cells formed more and significantly faster-growing organoids than the *LApc* cells (Fig. 4, L to N). However, during subsequent subculturing, the growth advantage of the *LApcL* cells was lost and the *LApc* and *LApcL* organoid growth rates and viability were similar during passages 3 to 8 (figs. S5, A to C, and S6, B and C).

To better understand which features of the *LApcL* adenomas are cancer cell–intrinsic and therefore preserved in the organoids, we confirmed *Lef1* deletion and then analyzed the *LApc* and *LApcL* organoids by scRNA-seq and immunohistochemistry for cell differentiation markers (fig. S5, D to J). Consistently with the similar organoid growth rates, the proportions of TA/proliferating cells and the *Ki67*^+^ cells were similar between the *LApcL* and *LApc* organoids (fig. S5H). Unlike in adenomas in vivo, the *LApcL* organoids showed a strong up-regulation of Lgr5 but no changes in Lyz1 expression when compared to the *LApc* organoids (fig. S5, D, E, and I). Moreover, *Muc2* RNA expression was down-regulated, whereas *ChgA* and *Villin1* (*Vil1*) expression was not altered after *Lef1* deletion (fig. S5E). Comparison of the scRNA-seqs of WT, *LApc*, and *LApcL* organoids indicated that mature enterocytes were decreased, whereas Wnt-high cells were increased after *Lef1* deletion (fig. S5H). However, similarly as in the intestinal tumors in vivo, the Wnt antagonists *Nkd1*, *Notum*, *Dkk3*, and *Axin2* were decreased by the *Lef1* deletion (fig. S5J).

To determine whether the growth of the *LApcL* organoids is cancer cell autonomous or dependent on external growth factors, we supplemented the organoid culture media with Wnt3a (W) alone for 5 days or together with epidermal growth factor (E), noggin (N), and Rspo1 (R) for three passages (fig. S6, A and D). We did not find any differences in the growth rates or the organoid forming capacities between the *LApc* and *LApcL* cells (fig. S6, B, C, E, and F). These results indicate that the in vitro culture conditions do not recapitulate the critical conditions that support the enhanced growth of the *LApcL* adenomas in vivo.

### *Lef1* deletion increases ectopic crypt formation and dedifferentiation in *Apc*-mutant adenomas

**Fig. 5.**
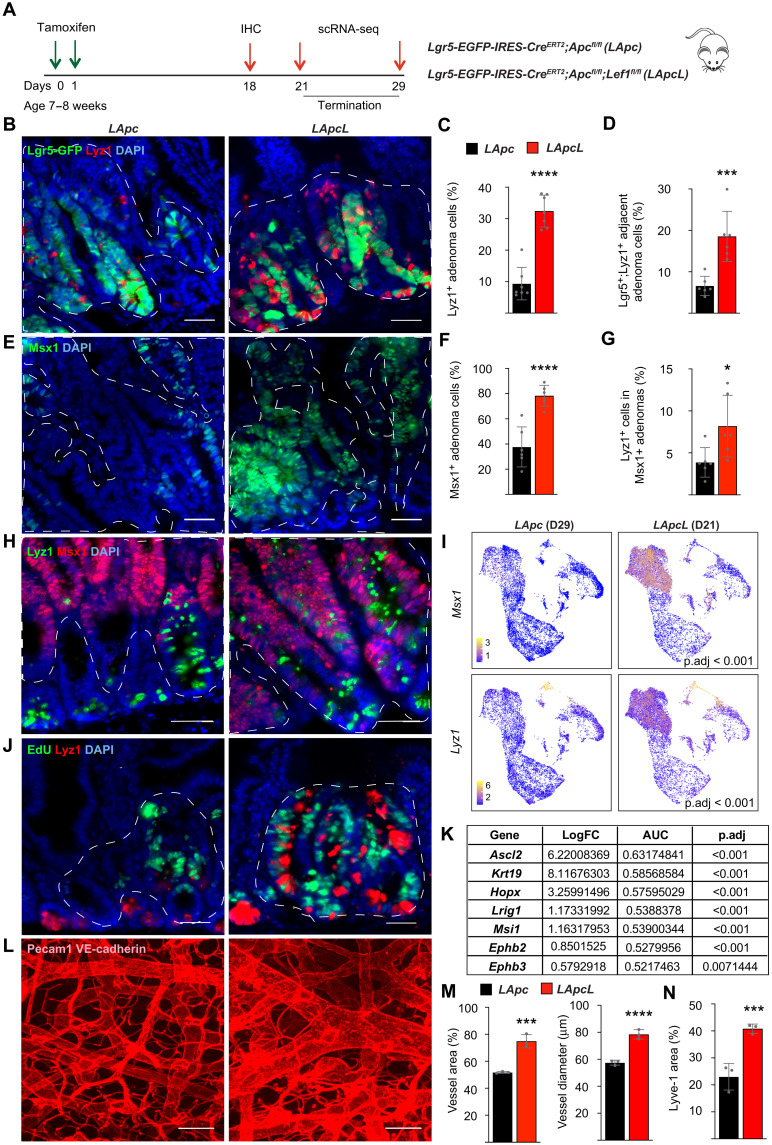
*Lef1* deletion increases the number of ectopic crypts in *Apc*-mutant adenomas. (**A**) Schematic of the experiment. (**B** to **D**) Lgr5-GFP and Lyz1 immunostaining, quantification of the percentage of (C) Lyz1^+^ cells and (D) Lgr5-GFP;Lyz1^+^ doublets in *LApc* and *LApcL* adenomas. Scale bars, 50 μm. *n* = 6 per group, ***P < 0.005 and ****P < 0.001. (**E** and **F**) Immunostaining (E) and quantification (F) of the ectopic crypt marker Msx1. Scale bars, 50 μm. *n* = 6 per group, ****P < 0.001. (**G** and **H**) Immunostaining (H) and quantification (G) of Lyz1^+^ cells in Msx1^+^ adenomas. Scale bar 50 μm. *n* = 6 per group, *P < 0.01. (**I**) UMAP visualization of the expression of *Msx1* and *Lyz1* in the scRNA-seqs from *LApc* and *LApcL* mice. (**J**) EdU and Lyz1 immunostaining. (**K**) Differential gene expression analysis of *Ascl2*, *Krt19*, *Hopx*, *Lrig1*, *Msi1*, *Ephb2*, and *Ephb3* in *LApcL* versus *LApc* adenoma cluster. (**L** and **M**) Immunofluorescent (L) staining and quantification (M) of Pecam1;VE-cadherin^+^ vessel area and diameter in *LApc* and *LApcL* intestines 21 days after tamoxifen. Scale bars, 50 μm. *n* = 3 per group, ***P < 0.005 and ****P < 0.001. (**N**) Quantification of Lyve-1 immunofluorescent staining in *LApc* and *LApcL* intestines 21 days after tamoxifen. *n* = 3 per group, ***P < 0.005. Data are shown as means ± SD. The dashed lines indicate nuclear β-catenin^+^ adenoma areas.

To study how the *Lef1*-deleted tumors fuel their growth despite the decreased number of Lgr5^+^ cells, we stained Lgr5-GFP^+^ crypts of *LApc* and *LApcL m*ice for Lyz1, which identifies ISC-sustaining Paneth cells (Fig. 5, A and B). We found a substantial increase in Lyz1^+^ cells and doublets of Lyz1^+^ and Lgr5^+^ cells that appeared to form intestinal crypt-like structures in β-catenin^+^ adenomas after *Lef1* deletion (Fig. 5, B to D). These results suggested that *Lef1* deletion increases the number of ectopic crypts in the adenomas. The number of cells expressing the ectopic crypt cell marker, Msh homeobox1 protein (Msx1) (*49*), was strongly increased in the *LApcL* adenoma areas (Fig. 5, E and F). Moreover, the Lyz1^+^ Paneth-like cells were much more frequent in the Msx1^+^ adenoma areas in the *LApcL* mice than in the *LApc* mice (Fig. 5, G and H). scRNA-seq analysis of the *LApc* and *LApcL* adenoma cells confirmed the increased expression of *Msx1* and *Lyz1* after *Lef1* deletion (Fig. 5I). We also found more cell proliferation in the proximity of the Lyz1^+^ cells in the *LApcL* intestines than in the *LApc* intestines, indicating that the ectopic crypt–like structures are associated with a zone of highly proliferating cells, as in the normal intestine (Fig. 5J). Consistent with this finding, *Ephb2* and *Ephb3*, which regulate the positioning and proliferation of ISCs, were increased after *Lef1* deletion (Fig. 5K) (*50*, *51*). Overall, these results indicate that in the *Apc*-deleted tumors, the *Lef1*-deficient cells assemble crypt-like structures, in which they up-regulate the expression of genes specific for the stem cell niche and cell proliferation. Furthermore, scRNA-seq analysis indicated that the Keratin19 (Krt19)^+^ radioresistant stem cell marker, the reserve stem cell markers *Hopx* and *Lrig1* and the *Ascl2* and *Msi1* transcripts that are required for the activation of the reserve stem cells were strongly increased in the *Lef1-*deleted adenoma cells (Fig. 5K) (*52*–*56*). These results suggest that *Lef1* deletion drives the plasticity and dedifferentiation of intestinal adenoma cells.

To further compare the vascular components of stem cell niches in *LApc* and *LApcL* mice, we performed immunostaining for platelet endothelial cell adhesion molecule 1 (PECAM-1), vascular endothelial (VE)–cadherin, and lymphatic vessel endothelial hyaluronan receptor 1 (Lyve-1) in the bottom of intestinal crypts. As expected by the increased tumor burden in the *LApcL* intestine, we found an expansion of blood and lymphatic vasculature after *Lef1* deletion (Fig. 5, L to N), indicating participation of the stromal microenvironment in the increased tumor growth.

**Fig. 6.**
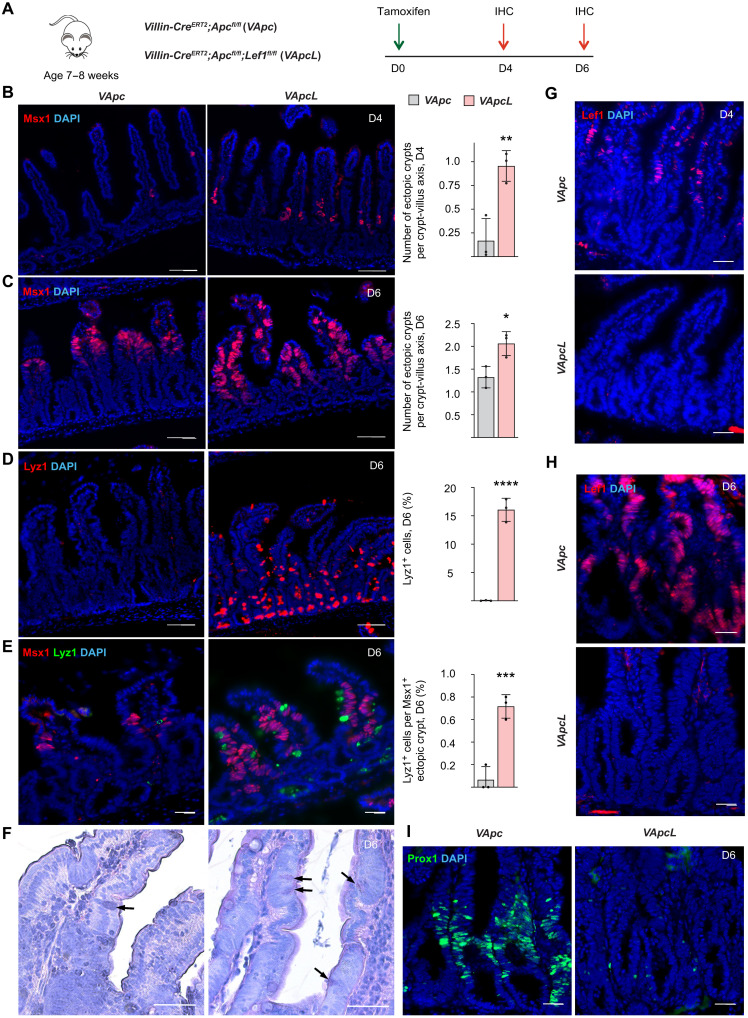
*Lef1* deletion increases the number of ectopic crypts in *Apc*-mutant adenomas. (**A**) *Villin-Cre^ERT2^;Apc^fl/fl^* (*VApc*) and *Villin-Cre^ERT2^;Apc^fl/fl^;Lef1 ^fl/fl^* (*VApcL*) mice received a single dose of tamoxifen at the age of 7 to 8 weeks, followed by immunohistochemistry analysis of the intestine 4 and 6 days thereafter. (**B** and **C**) Msx1 immunostaining and quantification of Msx1^+^ areas per crypt-villus axis in the intestines (B) 4 days and (C) 6 days after tamoxifen treatment. Scale bars, 50 μm. *n* = 3 per group, *P < 0.05 and **P < 0.01. (**D**) Lyz1 immunostaining and quantification of Lyz1^+^ cells in the intestine on day 6. Scale bars, 50 μm. *n* = 3 per group, ****P < 0.001. (**E**) Lyz1 and Msx1 immunostaining and quantification of Lyz1^+^ cells in Msx1^+^ ectopic crypt areas on day 6. Scale bars, 50 μm. *n* = 3 per group, ***P < 0.005. (**F**) HE (hematoxylin and eosin) images of *VApc* and *VApcL* intestines 6 days after tamoxifen. Arrows point to Paneth cells. Scale bars, 100 μm. (**G** and **H**) Lef1 immunostaining in the *VApc* and *VApcL* intestines (G) 4 days and (H) 6 days after tamoxifen treatment. Scale bars, 50 μm. (**I**) Prox1 immunostaining in the *VApc* and *VApcL* intestines 6 days after tamoxifen treatment. Scale bars, 50 μm. Data are shown as means ± SD. Each dot represents an average value analyzed from individual mouse.

Next, to study whether *Lef1* deletion can increase ectopic stem cell niches in another mouse model of intestinal adenomas, we deleted *Apc* with and without *Lef1* in all intestinal epithelial cells using the *Villin-Cre^ERT2^* allele (Fig. 6A). This induced rapid epithelial proliferation through the crypt-villus axis, leading to the death of the mice within 1 week. Immunohistochemical analysis confirmed the increased formation of ectopic crypts even at 4 days after *Apc* and *Lef1* deletion (Fig. 6B) and increased expression and coclustering of Msx1- and Lyz1-expressing cells 6 days after *Apc* and *Lef1* deletion (Fig. 6, C to E). Histological sections showed more ectopic crypts in the intestinal villi of the *Apc;Lef1*-deleted mice than the *Apc*-deleted mice (Fig. 6F). Lef1 expression was observed first in the villi 4 days after *Apc* deletion, and on day 6, Lef1 expression had extended throughout the intestinal epithelium (Fig. 6, G and H). At this time point, Prox1 staining was strongly down-regulated in the *Apc;Lef1*-deleted intestine (Fig. 6I).

### *Lef1* deletion increases tumorigenesis in *Apc^Min/+^* mice

**Fig. 7.**
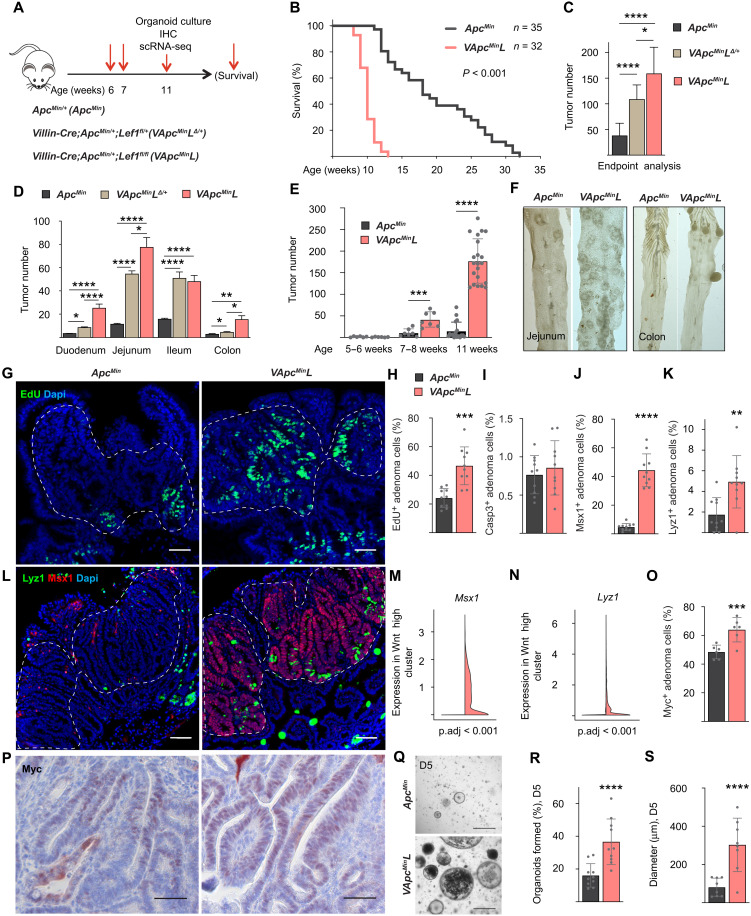
*Lef1* deletion increases tumorigenesis and decreases survival in *Apc^Min/+^* mice. (**A**) Schematic of the experiment. (**B**) Survival curves of *Apc^Min^* and *VApc^Min^L* mice. (**C** and **D**) Quantification of the total number of tumors (C) and the number of tumors in duodenum, jejunum, ileum, and colon (D) at termination. *Apc^Min^* (*n* = 35), *VApc^Min^L*^Δ*/+*^ (*n* = 15), and *VApc^Min^L* (*n* = 32). *P < 0.05, **P < 0.01, and ****P < 0.001. (**E**) The number of tumors at 6, 7, and 11 weeks of age. *n* = 6 to 7 mice per group at weeks 5 to 8. *n* = 18 (*Apc^Min^*) and *n* = 21 (*VApc^Min^L*) at week 11. ***P < 0.005 and ****P < 0.001. (**F**) Representative images of the jejunum and colon at week 11. (**G** to **I**) Immunostaining of EdU (G) and quantifications of EdU (H) and Casp3 (I) in adenoma cells. Scale bars, 50 μm. *n* = 10 mice per group, ***P < 0.005. (**J** to **L**) Lyz1 and Msx1 immunostaining (L) and quantification (J and K) in adenoma cells. Scale bars, 50 μm. *n* = 10 mice per group, **P < 0.01 and ****P < 0.001. (**M** and **N**) *Msx1* and *Lyz1* scRNA-seq analysis in the Wnt high clusters of *Apc^Min^* and *VApc^Min^L* adenomas. (**O** and **P**) Myc immunostaining (O) and quantification (P). Scale bars, 50 μm. *n* = 6 mice per group, ***P < 0.005. (**Q** to **S**) Representative images and quantifications of the (R) formation and (S) diameter of the *Apc^Min^* and *VApc^Min^L* organoids. Scale bars, 500 μm. *n* = 8 to 10 wells per group, ****P < 0.001. Data are shown as means ± SD. The dashed lines indicate nuclear β-catenin^+^ adenoma areas.

To analyze how longer-term *Lef1* deletion affects tumor development after a stochastic loss of the remaining WT *Apc* allele in *Apc^Min/+^* mice, we compared tumor growth in *Apc^Min/+^*(*Apc^Min^*), *Villin-Cre*;*Apc^Min/+^;Lef1^fl/+^*(*VApc^Min^L*^Δ*/+*^), and *Villin-Cre*;*Apc^Min/+^;Lef1^fl/fl^* (*VApc^Min^L*) mice (Fig. 7A). *Lef1* deletion in all intestinal epithelial cells led to a substantial decrease in the survival of *Apc^Min/+^-*mutant mice due to an increased tumor burden throughout the gut (Fig. 7, B to D). At termination, the heterozygous and homozygous *Lef1*-deleted mice had, respectively, 3.4- and 4.7-fold more macroscopic tumors than the *Apc^Min^* mice with WT *Lef1* (Fig. 7, C and D). The increase in tumor number in the small intestine and colon of *VApc^Min^L* mice was significant already at 7 weeks of age, and at the age of 11 weeks, the mice had over 11-fold more tumors than the *Apc^Min^* mice of the same age (Fig. 7, E and F).

scRNA-seq analysis of tumors of approximately equal size and location from 11-week-old *Apc^Min^* and *VApc^Min^L* mice showed that the proportion of Wnt-high cells was increased after *Lef1* deletion (fig. S7, arrow). At this time point, EdU incorporation and cleaved Casp3 staining indicated that *Lef1* deletion increases tumor cell proliferation, but not apoptosis (Fig. 7, G to I). Consistent with the results from the *LApcL* mice, Msx1, Lyz1, and Myc were increased after long-term *Lef1* deletion in the *Apc^Min^* mice (Fig. 7, J to P). Although the *VApc^Min^L* tumors were larger than the *Apc^Min^* tumors (fig. S8A), histopathological analysis showed no evidence of carcinomas or tumor invasion below muscularis mucosae (fig. S8). In histopathology of the tumors, both exhibited broad-based and sessile adenomas and atypical hyperplastic foci in the small intestine and broad-based adenomas in the colon (fig. S8, B to G). In the *VApc^Min^L* mice, tumors showed a sessile growth pattern more often than in the *Apc^Min^* mice. Villous structures were prevalent in the *VApc^Min^L* adenomas, but their occurrence per tumor was low. *VApc^Min^L* tumors grew in a more layered pattern, in which the basal layer was either unaffected or showed hyperplastic crypt epithelium (fig. S8, H and I). Single or aggregated Paneth cells were present in the basal and intermediate levels, and they were numerous in small adenomas of the distal jejunum and ileum of the *VApc^Min^L* mice (fig. S8I). The degree of dysplasia increased toward the luminal surface of the gut in the *VApc^Min^L* tumors, but not in the *Apc^Min^* tumors (fig. S8, K and L).

Next, we isolated cells from five to six intestinal tumors from 11-week-old *Apc^Min^* and *VApc^Min^L* mice and followed up their growth as organoids in Matrigel ex vivo. As expected by the high number of Wnt^+^ cells and ectopic crypts in the *VApc^Min^L* tumors in vivo, cells isolated from these tumors formed more organoids than cells from *Apc^Min^* tumors when similar numbers of cells were plated into three-dimensional (3D) culture (Fig. 7, Q to S).

To analyze whether Lef1 affects the tumor microenvironment, we performed scRNA-seq analysis of EpCAM^−^/CD45^−^ cells from the intestinal lamina propria of *Apc^Min^* and *VApc^Min^L* mice at the age of 10 weeks (fig. S9A). We did not detect significant differences in the relative proportions or in the differential gene expression of the *Pdgfra^+^* fibroblasts (FBs), *Acta2^+^* smooth muscle cells, or in the *Kcna1^+^* neuronal cells. However, the *VApc^Min^L* mice had proportionally more blood vascular endothelial cells, and lymphatic endothelial cells than the *Apc^Min^* mice (fig. S9, B and C). As in the *LApcL* mice (Fig. 5, L to N), immunostaining of PECAM-1, VE-cadherin, and LYVE-1 confirmed that blood vascular and lymphatic vessel areas and vessel diameters were increased after *Lef1* deletion in the *VApc^Min^L* mice (fig. S9, D and E), indicating stromal changes contributing to increased tumor growth.

## DISCUSSION

We show that deletion of *Lef1* in three different *Apc*-mutant intestinal cancer models accelerates tumor initiation and growth and concomitantly increases the expression of the Wnt-downstream targets Myc and Cd44. Unexpectedly, we found that six secreted Wnt antagonists (*Notum*, *Dkk2*, *Dkk3*, *Wif1*, *Axin2*, and *Nkd1*) were decreased when *Lef1* was codeleted with *Apc*, which may explain the widespread increase in cell proliferation in the tumors. Although *Lef1* deletion in the tumors decreased the number of Lgr5^+^ adenoma stem cells, it simultaneously increased the number of ectopic stem cell niches. The tumor promoting phenotype of *Lef1* deleted adenoma cells was not recapitulated in organoid cultures, indicating that the phenotype is not cell autonomous. Stromal and niche factors, such as increased vasculature, could contribute to the in vivo growth of *Lef1*-deleted adenomas; they remain to be analyzed in further studies. Furthermore, we found that in contrast to Wnt ligand–independent *Apc*-mutant adenomas, Lef1 was not expressed in Wnt ligand–dependent serrated adenomas. Consistent with our data indicating a role for Lef1 in suppressing ectopic crypt formation, the TSAs accumulate more ectopic crypts than conventional *Apc*-mutant adenomas (*57*).

We found that *Lef1* deletion in *Apc*-deficient tumor cells was associated with increased nuclear accumulation of β-catenin and up-regulation of its downstream targets Myc and Cd44, which have been considered as key mediators of intestinal tumorigenesis in the *Apc*-deficient cells (*10*, *39*). The critical role of Myc is evident from the study showing that *Myc* deletion rescues *Apc* deficiency in the small intestine (*10*). Even more notably, deletion of the upstream enhancer that controls Myc expression was sufficient to prevent Myc up-regulation and tumorigenesis in the *Apc^Min/+^* mice (*37*). Myc coordinates protein synthesis, cell growth, and tumorigenesis via regulation of ribosome biogenesis and translation (*41*, *42*). scRNA-seq analysis indicated that *Lef1* deletion also increased markedly transcripts encoding *Cd44*, which is a Wnt target, a CSC marker, and a key driver of intestinal tumorigenesis (*38*–*40*). *CD44* is induced in aberrant crypt foci in both humans and tumor-susceptible *Apc^Min/+^* mice, and its deletion in *Apc^Min/+^* mice inhibits the formation of aberrant crypt foci and intestinal tumorigenesis (*38*–*40*).

Although *Apc-*mutant adenomas are independent of Wnt ligands (*15*, *58*), we found that several Wnt antagonists were decreased after *Apc*;*Lef1* deletion. This observation may be functionally significant, as a recent study showed that the secreted Wnt antagonist Notum produced by the *Apc*-mutant adenomas inhibits Wnt signaling in the neighboring WT ISCs (*59*, *60*). We found that LEF1 expression was restricted to ligand-independent CRCs, whereas it was not expressed in the ligand-dependent CRCs. Ligand-dependent CRCs in humans depend on epigenetic down-regulation of WNT antagonists and a driver mutation in the *RNF43* or *RSPO* gene (*58*, *61*). In mice, loss of function of both *Rnf43* and *Znrf3* is necessary for the activation of Wnt signaling (*62*). We found that the *Rnf43*;*Znrf3*-mutant intestine expressed considerably less *Lef1* than the *Apc*-mutant intestine. Furthermore, *Rnf43* and *Znrf3* transcripts decreased significantly in the *Lef1*-deleted adenoma cells concomitantly with decreased expression of Wnt antagonists. These results suggest that *Lef1* deletion promotes growth of *Apc*-mutant adenomas in part by amplifying Wnt pathway activity in a ligand-dependent manner.

Ligand-dependent intestinal tumors consist almost entirely of Lgr5^+^ stem cells, and Lyz1^+^ Paneth cells that secrete Wnt3 (*62*). Wnt3 is not essential for ligand-independent intestinal adenomas (*63*) but is essential for *Rnf43;Znrf3*-mutant adenomas (*62*, *64*, *65*). We found that *Lef1* deletion increased Lyz1^+^ cells but, unexpectedly, decreased Lgr5^+^ stem cells in the *LApcL* adenomas. In the normal gut and adenomas, active, “working” Lgr5^+^ stem cells are located next to the Paneth cells, which provide essential niche factors for the stem cells (*3*, *5*, *66*). In comparison with the *LApc* adenomas, the Lyz1^+^ cells in the *LApcL* adenomas were associated with an increased number of proliferating adenoma cells and cells expressing the ectopic crypt marker Msx1, which is not expressed in WT intestine (*49*). We found that approximately 10 and 40% of Lgr5^+^ cells were located next to Lyz1^+^ cells in the *LApc* and *LApcL* adenomas, respectively, suggesting that the Lgr5^+^ ISCs give rise to the strongly increased ectopic crypts after *Lef1* deletion. *Lef1* deletion also increased the expression of *Myc*, *Cd44*, *Ephb2*, and *Ephb3*, which have been implicated in the proliferation and survival of intestinal stem and progenitor cells and in their positioning in along the crypt-villus axis (*39*, *50*, *51*, *67*, *68*). The modest decrease in transcripts encoding several Bmps in the *LApcL* adenoma cells may also be associated with the ectopic crypt phenotype as previous studies have shown that Gremlin1 can decrease Bmp signaling and promote ectopic crypt formation and expansion of Lgr5^−^ stem cells that function as cells of origin in *Apc*-mutant adenomas (*32*).

Our results suggest that the plasticity of intestinal cells is increased after *Apc;Lef1* deletion. *Lef1* deletion increased *Hopx* and *Lrig1* transcripts that mark reserve stem cells and renders Lgr5^+^ cells dispensable (*53*, *54*, *69*). These cells contribute to the plasticity of the intestinal epithelium as they can regenerate ISCs by dedifferentiation (*69*, *70*). Furthermore, *Lef1* deletion strongly increased expression of *Ascl2* and *Msi1*, which are critical for ISC regeneration (*55*, *56*). Overall, our results suggest that *Lef1* deletion favors dedifferentiation toward the ISC phenotype in the adenomas, and increases the number of ectopic crypts that are critical for CRC growth (*11*, *71*).

Some of the phenotypic properties of *Lef1*-deleted *Apc*-mutant adenomas resembled those described in *Apc;Kras*-mutant mice. Similar to *Lef1* deletion, oncogenic KRAS has been shown to induce *Myc* and *Cd44* expression and increase the dedifferentiation of the adenomas (*13*). Both mutant Kras and *Lef1* deletion also increased the formation of ectopic crypts with stem cell niches and activation of reserve stem cells (*16*, *57*, *71*, *72*). KRAS activation is known to confer a clonal advantage to *Apc*-mutant ISCs, which leads to crypt fixation and in increased tumor growth (*73*). Our finding that the number of aberrant crypt foci and adenoma cells was increased in the *LApcL* mice suggests that also *Lef1* deletion increases crypt fixation. Furthermore, after *Lef1* deletion, GSEA analysis showed increased activation of the KRAS downstream effectors *Pi3K/Akt/mTor* and *mTorc1* (*13*, *14*) and decreased TGFβ signaling, which is known to enhance dedifferentiation and accelerate tumorigenesis in the *Kras;Apc-*mutant intestine (*14*). Histopathological analysis showed that the *Lef1*-deleted adenomas had increased dysplasia, which is a known feature in *Kras*-mutant adenomas (*72*). Moreover, we found decreased LEF1 expression in the CMS3 and CRISA subtypes of CRC, which are characterized by KRAS mutations and in serrated adenomas, which typically harbor KRAS/Serine/Threonine-Protein Kinase B-Raf (BRAF) mutations (*30*, *31*, *57*). However, as we have analyzed here the function of Lef1 only in the *Apc*-mutant intestines, we cannot conclude whether Lef1 has a similar function in adenomas that have progressed with secondary mutations.

In our studies, we deleted both the dominant negative (dn) and full-length (fl) isoforms of *Lef1*. dn-Lef1, which cannot interact with β-catenin, inhibits transcription of Wnt target genes (*74*). Lef1 promoter 2, which is responsible for dn-Lef1 expression, is silent in CRC, whereas promoter 1 is a direct target of the Wnt pathway and thus activated in CRC (*74*). A balance between the two Lef1 isoforms thus seems critical for gut homeostasis. Although LEF1 is almost always expressed in CRC and although normal intestinal crypts depend on Wnt signals, LEF1 transcription is not activated in normal crypts. It would thus be important to find out how the LEF1 locus becomes accessible to aberrant Wnt signals in CRC.

Perhaps, because of functional redundancy between members of Tcf/Lef family, we detected compensatory increase in *Tcf7* or *Tcf7l2* transcripts in the *Lef1* deleted adenoma cells. fl-Tcf4 and dnTcf7 transcripts are the predominant Tcf/Lef1 family isoforms expressed in the intestine, which explains the opposite intestinal phenotypes in mice deleted of Tcf7l2 (lack of cycling stem cells) versus Tcf7 (intestinal polyposis) (*8*, *21*, *22*). It is possible that the increased expression of *Tcf7* and *Tcf7l2* contributes to the phenotype of the *Lef1* deleted mice. Members of the Tcf/Lef1 family can act as transcriptional activators or repressors, depending on their interacting genes and cell types in which they are expressed. An interesting recent study shows that the intrinsic histone deacetylase (HDAC) activity of Tcf1 and Lef1 can restrain the chromatin accessibility of genes that encode coinhibitory receptors in T cell activation (*75*). Additional studies are needed to show whether *Lef1* deletion phenotype of is caused by DNA sequence-specific modulation of transcriptional initiation or by modulation of chromatin accessibility via corepressors or the intrinsic HDAC activity. Future studies should also address the metabolic and possible immune functions of *Lef1* in intestinal tumors.

In summary, we show that Lef1 operates a negative feedback loop in *Apc-*mutant cancer cells that limits tumor initiation and progression by restricting tumor cell dedifferentiation and expression of key downstream effectors, such as the Myc oncogene, and by reducing the formation of ectopic stem cell niches. On the basis of the similarities of *Lef1*-deficient *Apc*-mutant adenomas with *Kras*-mutant CRC, we propose that Kras and Lef1 act in the same pathway in the process of transformation of ISCs to promote (Kras) or reduce (Lef1) the fixation of mutated crypts. Our finding of extremely low Lef1 expression in ligand-dependent CRCs also argues that Lef1 levels in CRC tumor cells could serve as a biomarker for the identification of patients with CRC who may benefit from WNT ligand inhibition.

## MATERIALS AND METHODS

### Animal experiments

Animal experiments were approved by the National Animal Experiment Board of Finland. Mice were housed and monitored according to the Federation of European Laboratory Animal Science Associations guidelines and recommendations. The mice were weighed and their health was closely monitored during the experiment. The mouse lines *Lef1^fl/fl^* (*25*), *Apc^fl/fl^* (*76*), *Lgr5-EGFP-IRES-Cre^ERT2^* (*4*), *Rosa26^LSL-TdTomato^* (Jackson Laboratory, stock no. 021875), *Apc^Min/+^* (Jackson Laboratory, stock no. 002020), *Villin-Cre^ERT2^* (Jackson Laboratory, stock no. 020282), and *Villin-Cre* (Jackson Laboratory, stock no. 021504) have been described previously. *Lef1^fl/fl^* mice with mixed C57BL/6 and 129SV background were used after backcrossing to the C57BL/6 strain for >6 generations. All experiments were performed three times with independent cohorts. Approximately equal numbers of male and female mice of same age were used for all the experiments.

### WT and Lef1^fl/fl^ mice

For induction of Cre^ERT2^-mediated recombination, mice received a single dose of tamoxifen (Sigma-Aldrich, #T5648; dissolved in corn oil at 2 mg) by gavage. Mice were injected with EdU (Invitrogen, #A10044; dissolved in 0.9% saline at 1 mg) by intraperitoneal injection 2 hours before their termination. For analysis of epithelial regeneration after radiation damage, mice were exposed to γ-irradiation from a caesium-137 source at 0.423 Gy/min with a single dose of 10 Gy.Apc^fl/fl^;Lgr5-EGFP-IRES-Cre^ERT2^ and Apc^fl/fl^;Lef1^fl/fl^;Lgr5-EGFP-IRES-Cre^ERT2^ mice received two doses of tamoxifen by gavage during consecutive days at the age of 7 to 8 weeks. EdU was injected 4 hours before termination. For the survival analysis of the mice, the termination criteria were determined as weight loss (>10%), blood in feces or worsening under general condition. Apc^fl/fl^;Villin-Cre^ERT2^ and Apc^fl/fl^;Lef1^fl/fl^;Villin-Cre^ERT2^ mice received a single dose of tamoxifen.Apc^Min/+^ and Apc^Min/+^;Lef1^fl/fl^;Villin-Cre mice were injected with EdU 4 hours before termination. Apc^fl/fl^;Villin-Cre^ERT2^ and Rnf43^fl/fl^;Znrf3^fl/fl^;Villin-Cre^ERT2^ mice received a single dose of tamoxifen by gavage.

### Immunohistochemistry and in situ hybridization

The intestines were fixed with 4% paraformaldehyde (PFA; Histolab, HL95753.1000) overnight (o/n), washed with phosphate-buffered saline (PBS), dehydrated, embedded into paraffin, and cut into 5-μm sections, which were deparaffinized and subjected to heat-induced target retrieval. Nonspecific binding of the antibodies was blocked with 0.1 M tris-HCl, 0.15 M NaCl, and 0.5% TSA-blocking buffer. For peroxidase staining, endogenous peroxidase activity was blocked with 3% hydrogen peroxide-methanol incubation.

For whole mount staining of the vessels, mice were euthanized and perfused with ice-cold PBS for 5 min. Intestines were washed with PBS, flushed with 4% PFA (house-made), followed by PFA fixation at +4°C o/n. Intestines were then washed with PBS, and 1-cm pieces from the same segment of the gut were permeabilized with 0.3% Triton X-100–PBS (Tx-PBS) for 2 hours, blocked with 5% normal donkey serum, 0.2% bovine serum albumin (BSA), 0.05% NaN_3_, and 0.3% Tx-PBS. After incubation with primary antibodies for 3 days, the intestinal segments were washed with 0.3% Tx-PBS, incubated with secondary antibodies for 24 hours, and washed again with 0.3% Tx-PBS. Pieces were postfixed with 1% PFA for 5 min, rinsed with PBS, and stained with 4′,6-diamidino-2-phenylindole (DAPI).

For staining of the organoids, the *LApc* and *LApcL* organoids were incubated with 10 μM EdU (Invitrogen, A10044) for 30 min at +37°C, fixed with 4% PFA for 30 min at room temperature (RT), washed with PBS, and blocked for 1 hour in 0.3% Triton X-100, 0.5% BSA, and 5% horse serum. Incubation with the Lyz1 antibody (1:300) was at 4°C o/n. EdU labeling was detected according to the manufacturer’s instructions (Invitrogen, C10337). Organoids were washed extensively with 0.3% Triton X-100 in PBS and incubated with Alexa Fluor donkey anti-rabbit 647 (1:500; Invitrogen) at RT for 2 hours. After extensive washes in 0.3% Triton X-100 in PBS, the organoids were incubated with DAPI (2.5 μg/ml) at RT for 10 min and washed with PBS. For 2D staining, the fixed organoids were embedded into 2% agarose gel, embedded in paraffin blocks, deparaffinized, and cut into 5-μm sections.

The following antibodies were used for the immunostainings: rabbit anti-Lef1 (1:200; Cell Signaling Technology, #2230, C12A5), rabbit anti-GFP (1:1000; Torrey Pines Biolabs, TP401), chicken anti-GFP (1:200; Abcam, ab13970), mouse anti–β-catenin (1:200; BD Biosciences, 610153), goat anti-Prox1 (1:300; R&Systems, AF2727), mouse anti–E-cadherin (1:400; BD Biosciences, 610181), rabbit anti-Mucin2 (1:300; Santa Cruz Biotechnology, sc-15334), rabbit anti–Chr-A H-300 (1:300; Santa Cruz Biotechnology, sc-13090), rabbit anti-DCAMKL1 (1:300; Abcam, ab31704), rabbit anti-Lyz1 (1:500; Dako, A0099), goat anti-Msx1 (1:200; R&D Systems, AF5045), rabbit anti-cleaved Casp3 (1:400; Cell Signaling Technology, #9661), rabbit anti-Myc (1:200; Abcam, ab32072), rat anti-CD44 (1:500; BioLegend, no. 103066), goat anti-Sox17 (1:400; R&D Systems, AF1924), allophycocyanin (APC) anti-mouse Cd24 (1:200; BioLegend, #101814), rabbit anti–LYVE-1 (1:1000; house-made), goat anti–VE-cadherin (1:500; R&D Systems, AF1002), and goat anti-CD31/Pecam (1:100; R&D Systems, AF3628).

For immunofluorescent staining, Alexa Fluor 488–, Alexa Fluor 594–, and Alexa Fluor 647–conjugated secondary antibodies (1:500; Invitrogen) were used, and nuclei were counterstained with DAPI containing VECTASHIELD mounting medium (Vector Laboratories, H-1200). The Click-IT EdU Alexa Fluor 488 Imaging Kit (Invitrogen, C10337) was used for EdU detection according to the manufacturer’s instructions. Images were captured with Zeiss Axio Imager Z2 microscope or with Zeiss LSM780 or LSM880 confocal microscope.

For peroxidase staining, ImmPRESS horseradish peroxidase (HRP) secondary antibodies (Vector Laboratories, MP-7401 and MP-7402) were used, and 3-amino-9-ethylcarbazole (Sigma-Aldrich, A5754) was used for HRP detection. Peroxidase staining was counterstained with hemalum. Slides were scanned with Pannoramic 250 Flash II, 3DHISTECH. For analysis and quantification of the images, Fiji 1 (Fiji Is just ImageJ) was used.

Lef1 (Advanced Cell Diagnostics, #441868) mRNA hybridization was performed using the RNAscope 2.5 LS Reagent Kit–BROWN (Advanced Cell Diagnostics) on a BOND RX autostainer (Leica) according to the manufacturer’s instructions. Positive control probes (Mm-Ppib; Advanced Cell Diagnostics, #313918) were included in each run to ensure RNA integrity and staining specificity.

### Organoid culture experiments

*LApc* and *LApcL* mice received two doses of tamoxifen, and their intestinal crypts were isolated 17 or 21 days thereafter. One thousand single cells isolated from *LApc* or *LApcL* intestines were embedded per well in Matrigel [Growth factor reduced (GFR), phenol free; BD Biosciences, #356231]. Organoids were cultured in advanced Dulbecco’s modified Eagle’s medium (DMEM)/F12 (Gibco, #12634-010) with 10 mM Hepes (Thermo Fisher Scientific, #15630106), glutamine, penicillin/streptomycin, 1× B27 supplement (Gibco, #17504-044), and 1× N2 supplement (Gibco, #17502-048). Organoids were cultured without growth factors for two passages. At passage 3, organoid culture medium was supplemented with the indicated combinations of recombinant Wnt3a (100 ng/ml; R&D Systems, 1324-WN), recombinant human R-spondin1 (RSPO1) (1 mg/ml; R&D Systems, 4645-RS), recombinant murine noggin (100 ng/ml; PeproTech, 250-38), and/or recombinant mouse epidermal growth factor (50 ng/ml; Gibco, PMG8041). Fresh medium was changed every 2 days. Organoids were supplemented with Wnt3a for 1 week or with different growth factor combinations for three passages (15 to 18 days). During subculture, the cells were dissociated into small cell clusters, and similar numbers of *LApc* and *LApcL* cells were plated in Matrigel. For analysis of the organoid forming capacity at passage 5, the cell clusters were dissociated into single cells and 1000 viable cells were embedded in Matrigel.

For *Apc^Min^* and *VApc^Min^L* organoid cultures, 1000 single cells dissociated from *Apc^Min^* and *VApc^Min^L* tumors were embedded per well in Matrigel (GFR, phenol free; Corning, #356231). Organoids were cultured in advanced DMEM/F12 (Gibco, #12634-010) with 10 mM Hepes (Thermo Fisher Scientific, #15630106), glutamine, penicillin/streptomycin, 1× B27 supplement (Gibco, #17504-044), and 1× N2 supplement (Gibco, #17502-048).

### Dissociation of intestinal epithelial cells, lamina propria, intestinal tumors, and organoids into single cells

Small intestines of WT, *LApc*, and *LApcL* mice were isolated and washed with cold PBS, cut into three to four pieces, and incubated in 10 mM EDTA in an orbital shaker at +4°C for 30 min. Tissues were washed with PBS, and cells from the luminal surface were scraped off with a glass slide. The cells were then centrifuged at 300 rpm at +4°C followed by incubation with dispase II (4 mg/ml), collagenase I (1 mg/ml), collagenase H (1 mg/ml), and deoxyribonuclease I (DNase I) (1 U/ml) for 7 min at 32°C; washed with DMEM with 2% fetal bovine serum (FBS); and centrifuged at 300 rpm at +4°C for 2 min, after which floating cells were collected and filtered through a 40-μm mesh. The remaining cells were centrifuged at 300 rpm at +4°C for 5 min and incubated in trypsin-EDTA with DNase I (1 U/ml) for 5 min at 32°C. After a wash with DMEM with 2% FBS, the cells were centrifuged at 300 rpm at +4°C and filtered through a 40-μm mesh.

For tumor dissociation, 3 to 10 tumors of approximately same size and from similar locations in the intestines were removed from 11-week-old *Apc^Min^* and *VApc^Min^L* mice. Tumors were cut into small pieces and incubated with enzymes (dispase II, 4 mg/ml; collagenase I, 1 mg/ml; collagenase H, 1 mg/ml; and DNase I, 1 U/ml) for 30 min at +32°C. Tumor cells were then washed with DMEM with 2% FBS and incubated with trypsin-EDTA for 15 min at +37°C. Stromal cell isolation from lamina propria of 11-week-old *Apc^Min^* and *VApc^Min^L* was performed as previously described (*77*). WT, *LApc*, and *LApcL* organoids were trypsin treated to obtain single cells. Cells were washed two times with DMEM with 2% FBS and filtered through 40-μm mesh.

### Fluorescence-activated cell sorting

Epithelial cells from WT, *LApc*, and *LApcL* mice and stromal cells from *Apc^Min^* and *VApc^Min^L* mice were sorted on BD Influx (BD Biosciences) for scRNA-seq analysis and gene expression validation. The epithelial cells were surface-stained with the following antibodies at 1:500 dilution for 30 min on ice: anti-Mo CD326 (EpCAM) phycoerythrin (eBioscience; Invitrogen, #12-5791-82), CD16/CD32 (mouse BD Fc block; BD Pharmingen, #553141), anti-CD45 (eBioscience, #48-0451-82), and anti–Ter-119 (eBioscience, #48-5921-82). Sorted cells were resuspended into Hanks’ balanced salt solution with 0.04% BSA.

### scRNA-seq and data analysis

All samples were analyzed using the Chromium Single-Cell 3′ RNA-sequencing platform (10x Genomics, Pleasanton, CA) with the Reagent Kit v2 or v3 according to the manufacturer’s instructions. Sample libraries were sequenced using the Illumina NovaSeq 6000 system (50,000 reads per sample). BCL files were converted to FASTQ, and reads were quantified with Cell Ranger 2.1.1 using the prebuilt reference provided by 10x Genomics (refdata-cellranger-mm10-1.2.0). Unless otherwise stated, we used the Seurat R package 3.1.1 for data analysis. We excluded outlier cells (top and bottom, 1%) based on total number of genes detected and low-quality cells with more than 20% Unique Molecular Identifiers (UMIs) corresponding to mitochondrial genes. We then identified potential doublets using scDblFinder 1.1.8 from Bioconductor. UMI counts for each cell were divided by total counts for that cell, multiplied by 10,000 as scaling factor, and natural log-transformed to obtain normalized data. To identify variable features while controlling for the mean-variance relationship in the data, we used the FindVariableFeatures functionality from Seurat, with selection.method set to mean.var.plot (mvp). To integrate cells across different samples and adjust for potential batch effects, we used the canonical correlation analysis–based approach implemented in Seurat. Briefly, this approach first identifies “anchors,” i.e., shared cell states, between pairs of datasets using variable features. These anchors are then used to harmonize the datasets. Having obtained an integrated dataset for downstream analysis, we further reduced the dimensionality of the data by retaining the top 20 principal components (PCs) of the anchoring features, which was subsequently used as input for 2D visualization by Uniform Manifold Approximation and Projection (UMAP) using the default setting in Seurat. To identify discrete cell populations, we performed graph-based clustering in Seurat and used the Louvain algorithm with the resolution parameter set to 0.2 on the shared nearest-neighbor graph that was constructed from top 20 PCs by setting the k.param to 20. Gene module scores were computed per cell using the AddModuleScore functionality of Seurat. Briefly, normalized features were binned into 24 bins based on averaged expression. The module scores were then computed by calculating the average expression of genes belonging to the module and subtracted by the average expression of 100 control genes randomly selected from each bin. We used default plotting functionalities of Seurat to visualize data and occasionally adapted the output using ggplot2. Cell type annotations were assigned to each cluster based on module enrichment scores computed with marker gene signatures obtained from a previously annotated reference (*35*). To distinguish normal cells from the adenoma cells within the Wnt/TA cell population, we used the approach implemented in the batchelor package from Bioconductor (*78*). Briefly, we first used the multiBatchNorm functionality to remove systematic differences in coverage (aka sequencing depth) across samples. Next, we applied the fastMNN functionality, with top 20 PCs and the number of nearest neighbors set to 53, to correct for potential batch effects using the mutual nearest neighbor (MNN) approach of Haghverdi *et al.* (*78*). For differential expression analysis, we used the Presto R package, providing a fast implementation of rank-sum and auROC analyses. Given the large sample sizes in scRNA-seq, the *P* values are often spuriously small. We therefore used the area under the curve (AUC) as computed by Presto, as gene ranking metric in downstream GSEA, or along with log fold changes to create volcano visualization of differentially expressed genes. Intuitively, the AUC is a measure of separation between the two groups, with an AUC value of 0 or 1 indicating perfect separation and with an AUC value of 0.5 implying lack of predictive power to separate the two groups. We used the fgsea implementation of GSEA from Bioconductor to look for the enrichment of hallmark pathways obtained from the Broad Institute’s MSigDB database.

The final dataset of WT, *LApc*, and *LApcL* epithelial cells consisted of 10,044, 9651, and 14,062 cells, respectively. One mouse per group was used in the experiment, which was repeated twice.

The final dataset of *Apc^Min^* and *VApcMinL* adenomas consisted of 9099 and 9890 cells, respectively. Three separate experiments were integrated in the final dataset. The Seurat R package 3.1.1 was used for analysis of the data (*79*). We excluded low-quality cells with more than 20% UMIs corresponding to mitochondrial genes. All datasets were processed with default settings for integration anchors. UMAP plots and list of differentially expressed genes were generated as instructed by the Seurat website. Cluster identification was performed using the previously published intestinal cell markers (*35*).

For *Apc^Min^* and *VApcMinL* adenomas, the following marker genes were used for the heatmap (fig. S4):

**Table:** 

**Enterocytes**	*Apoa1*, *Rbp2*, *Fabp1*, *Prap1*, *Apoc3*, *Aldob*, *Mttp*, *Krt20*, *Alpi*, *Gsta1*
Wnt proliferation	*Nkd1*, *Notum*, *Prox1*, *Cd44*, *Lgr5*, *Ccnd1*, *Myc*, *Tubb5*, *Ptma*, *Birc5*, *Mki67*, *Hspd1*, *Ube2c*, *Hmgb2*, *Top2a*
T cells	*Cd3g*, *Cd3e*, *Cd7*, *Nkg7*, *Cd8a*, *Gnly*
Macrophages	*C1qa*, *C1qb*, *Il1b*, *Cxcl2*, *Mpeg1*
B cells	*Jchain*, *Mzb1*, *Cd79a*, *Iglc2*, *Vpreb3*
Fibroblasts	*Pdgfra*, *Hhip*, *Bmp4*, *Vim*, *Des*, *Dcn*, *Col1a1*, *Col1a2*
Paneth/goblet	*Gsta1*, *Zg16*, *Tff3*, *Muc2*, *Agr2*, *Lyz1*, *Klk1*, *Spdef*
Endothelial	*Pvalp*, *Pecam1*, *Cd34*, *Flt1*, *Ptprb*, *Ly6a*

The final dataset of *Apc^Min^* and *VApcMinL* lamina propria consisted of 4753 and 6007 cells respectively.

### RNA extraction, cDNA synthesis, and quantitative polymerase chain reaction

Organoid RNA was isolated using the NucleoSpin RNA isolation kit (Macherey-Nagel, 740955.50) according to the manufacturer’s instructions. The High-Capacity cDNA Reverse Transcription Kit (Thermo Fisher Scientific, 4368814) was used for cDNA synthesis according to the manufacturer’s instructions. Maxima SYBR Green/ROX qPCR Master Mix (K0221, Thermo Fisher Scientific) and a BioRad real-time PCR instrument CFX96 were used for quantitative reverse transcription polymerase chain reaction (qPCR). *C*_t_ values were normalized to those for hypoxanthine phosphoribosyltransferase 1 (Hprt1) using the ΔΔ*C*_t_ method. Statistical analyses were performed with Student’s *t* tests.

The following primers were used in qPCR: *Lef1*, 5′-GCATGAACAGAGAAAGGAGCA-′3 (forward) and 5′-ATTTAGCCTGCTCTTCACGG-′3 (reverse); *Lgr5*, 5′-CCTGTCCAGGCTTTCAGAAG-′3 (forward) and 5′-CTGTGGAGTCCATCAAAGCA-′3 (reverse); *Lyz1*, 5′-GAGACCGAAGCACCGACTATG-′3 (forward) and 5′-CGGTTTTGACATTGTGTTCGC-′3 (reverse); *Muc2*, 5′-TGTGGAACCGGGAAGATG-′3 (forward) and 5′-GACCACAGGTATGGTTCTGGA-′3; *ChgA*, 5′-CGATCCAGAAAGATGATGGTC-′3 (forward) and 5′-CGGAAGCCTCTGTCTTTCC-′3 (reverse); *Vil1*, 5′-GCTTGCCACAACTTCCTAAGAT-′3 (forward) and 5′-TCAGTTTAGTCATGGTGGACGA-′3 (reverse); *Hprt1*, 5′-GGGGACATAAAAGTTATTGGTGG-′3 (forward) and 5′-AACCAGGGAAAGCAAAGTTTG-′3 (reverse).

### Analysis of human RNA data

The analysis was performed on previously published polyp RNA-seq data (*58*). In addition to the transcriptomic data, information about the mutation status for the Wnt driver genes was obtained. The transcriptomic data were processed with EdgeR R package to obtain normalized counts per million (CPM). The expression of the LEF1 gene was assessed by analysis of the samples based on WNT alteration and histological class. Graphs were plotted using ggplot2 in R.

Lef1 expression in human CRC subtypes was assessed as previously described (*80*). Briefly, processed microarray data were obtained from Gene Expression Omnibus under the accession ID GSE39582. Metadata including CMS classification labels and CRISs were separately obtained as previously reported. After excluding 75 Microsatellite instable (MSI) samples, 444 Microsatellite stable (MSS) CRC samples were retained for the analysis of Lef1 expression.

### Statistical analysis

Statistical analysis of two groups was performed with Student’s two-tailed *t* test. Statistical analysis of three groups was performed using analysis of variance (ANOVA). *P* < 0.05 was considered as statistically significant and the significance is marked by *P < 0.05, **P < 0.01, ***P < 0.005, and ****P < 0.001. Values are represented as means ± SD or SEM as indicated in the figures.

## Supplementary Materials

### Other Supplementary Material for this manuscript includes the following:

Tables S1 and S2

Correction 20 May 2022:
Due to a rendering error, the axes for Figures S4(A), S5(H) and S9(B) were illegible. The supplementary materials PDF has now been corrected. The original version is available here:

## Notes

View/request a protocol for this paper from *Bio-protocol*.
