# Supplementary material for: Lef1 restricts ectopic crypt formation and tumor cell growth in intestinal adenomas
Source: Sci Adv. 2021 Nov 17;7(47):eabj0512. doi: 10.1126/sciadv.abj0512 (PMC8598008; doi:10.1126/sciadv.abj0512)
Supplement: Supplementary file 1 — Figs. S1 to S9 Legends for tables S1 and S2 [file sciadv.abj0512_sm.v2.pdf]

CORRECTED 20 MAY 2022; SEE FULL TEXT

Science Advances
AAAS

Supplementary Materials for

Lef1 restricts ectopic crypt formation and tumor cell growth in intestinal adenomas

Sarika Heino, Shentong Fang, Marianne Lähde, Jenny Högström, Sina Nassiri, Andrew Campbell, Dustin Flanagan, Alexander Raven, Michael Hodder, Nadia Nasreddin, Hai-Hui Xue, Mauro Delorenzi, Simon Leedham, Tatiana V. Petrova, Owen Sansom, Kari Alitalo*

*Corresponding author. Email: kari.alitalo@helsinki.fi

Published 17 November 2021, *Sci. Adv.* **7**, eabj0512 (2021)
DOI: 10.1126/sciadv.abj0512

The PDF file includes:

Figs. S1 to S9
Legends for tables S1 and S2

Other Supplementary Material for this manuscript includes the following:

Tables S1 and S2

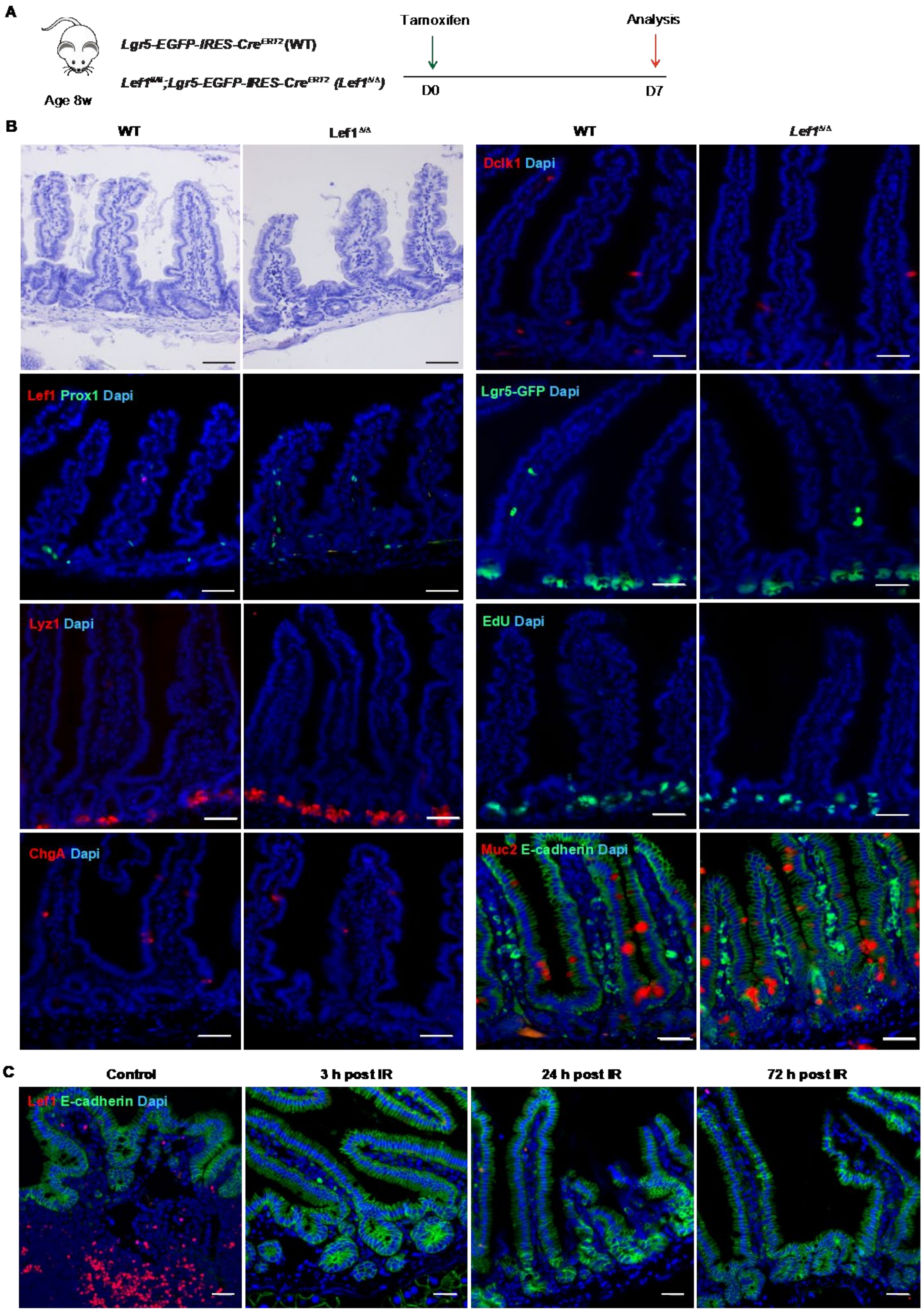

Heino et al., Fig S1

Figure S1. Lef1 is not required for homeostasis or regeneration of the healthy intestine.

(**A**) Lgr5-EGFP-IRES-Cre^{ERT2} (WT) and Lgr5-EGFP-IRES-Cre^{ERT2};Lef1$^{fl/fl}$ (Lef1$^{\Delta/\Delta}$) mice received a single dose of tamoxifen at the age of 8 weeks and were analyzed one week thereafter.

(**B**) Hemalum staining and staining for Lef1, Prox1, Lysozyme1 (Lyz1), Chromogranin A (ChgA), Doublecortin like kinase 1 (Dclk1), Lgr5-GFP, EdU, Mucin2 (Muc2) and E-cadherin in WT and Lef1$^{\Delta/\Delta}$ intestine. Scale bars 50µm, n= 3 mice per group.

(**C**) Lef1 and E-cadherin staining in WT intestine at 3, 24 and 72 hours after 10 Gy irradiation. Scale bars 20 µm, n= 3 mice per time point. Lef1 expression in the T-cells of Peyer's patches was used as a positive control for Lef1 staining.

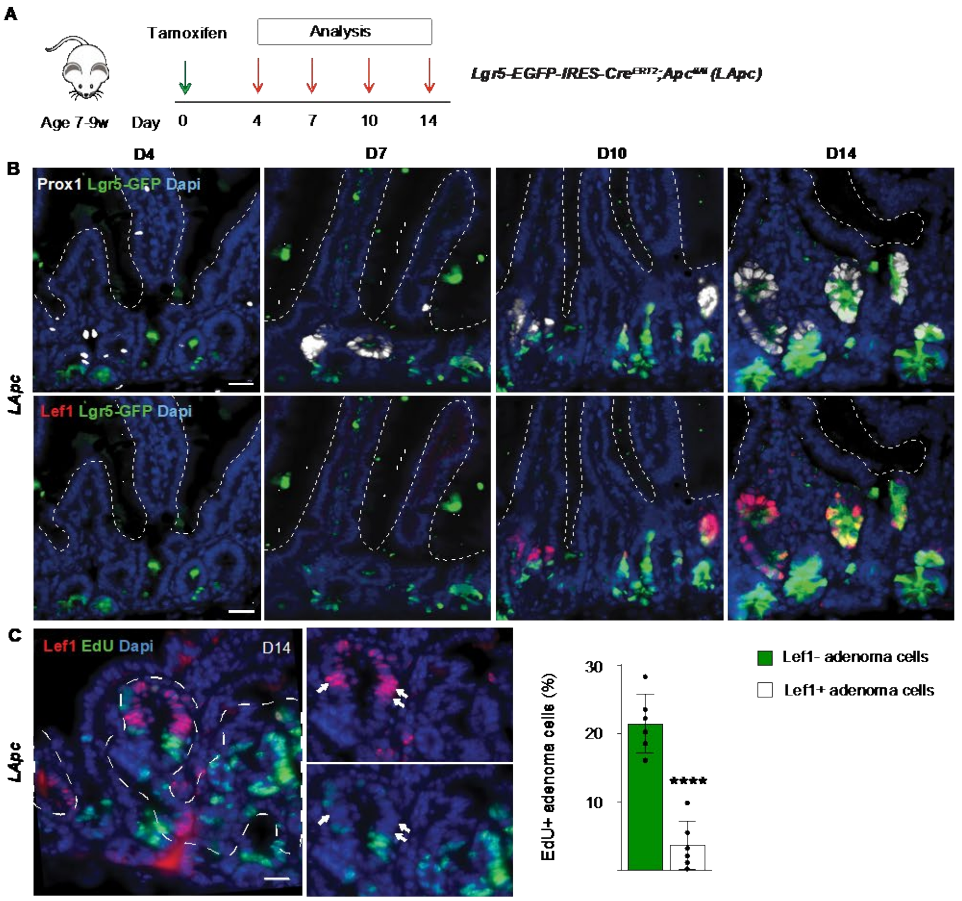

Heino et al., Fig. S2

Figure S2. Lef1 is induced in Lgr5+;Prox1+ cells after *Apc* deletion.

(A) Schematic of the experiment. *Lgr5-EGFP-IRES-Cre*^{ERT2}*;Apc*$^{fl/fll}$ *(LApc)* mice received a single dose of tamoxifen at the age of 7-9 weeks and were analyzed at the time points indicated.

(B) Immunofluorescent staining of Prox1, Lef1 and Lgr5-GFP 4, 7, 10 and 14 days after tamoxifen administration. Dashed line indicates the luminal surface of the intestine. Scale bar 20 µm, n=3 per time point.

(C) EdU and Lef1 staining and quantification 14 days after *Apc* deletion. Dashed line indicates nuclear β-catenin+ area. Arrows point Lef1+EdU- cells. Scale bar 20 µm, n=6, P****<0.001. Data are shown as mean±SD.

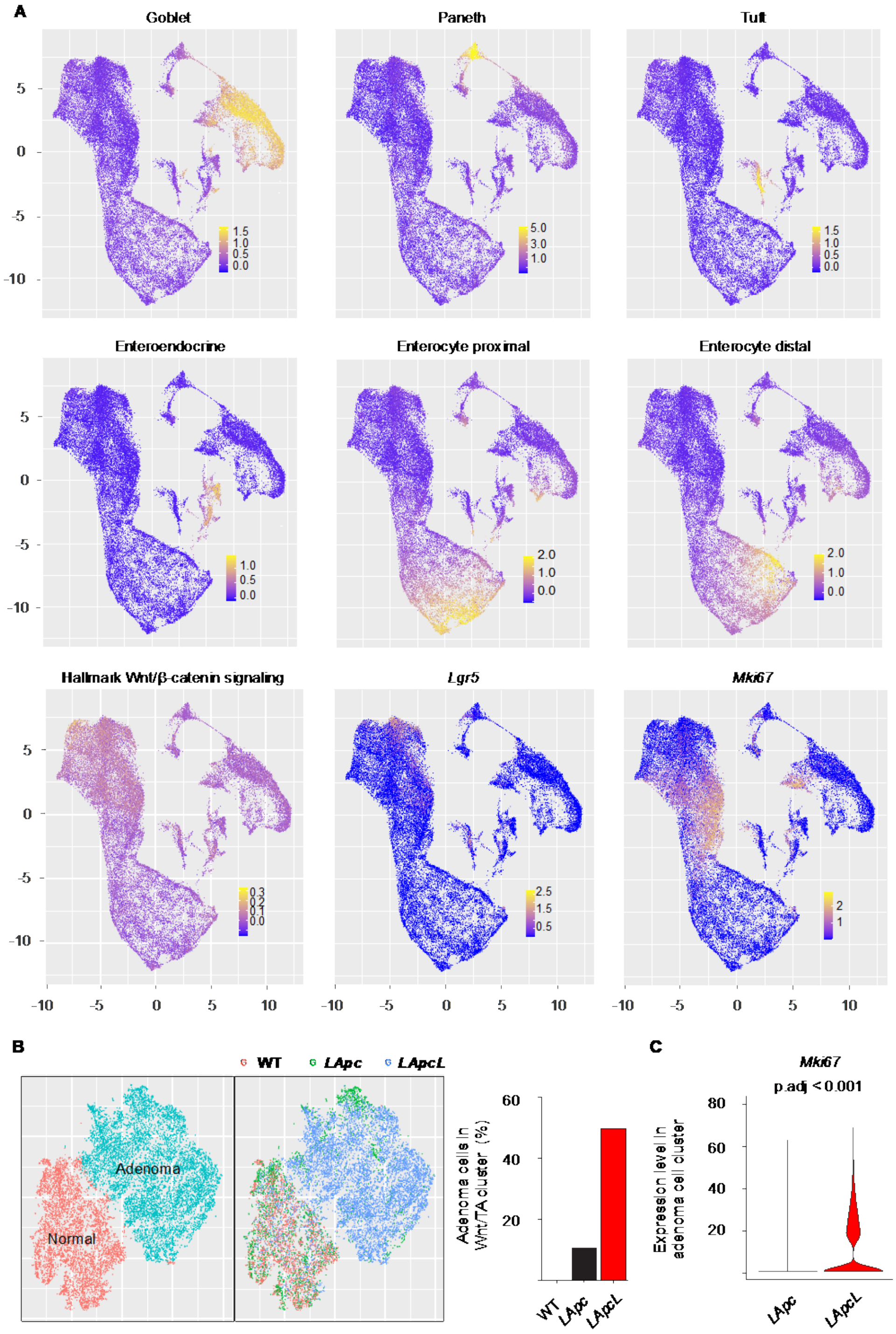
A
Goblet
Paneth
Tuft
Enteroendocrine
Enterocyte proximal
Enterocyte distal
Hallmark Wnt/β-catenin signaling
Lgr5
Mki67
B
WT
LApc
LApcL
Adenoma
Normal
Adenoma cells in Wnt/TA cluster (%)
C
Mki67
p.adj < 0.001
Expression level in adenoma cell cluster

Fig. S3. Cluster identification among intestinal EpCAM+ single cells

(**A**) Module enrichment scores of signatures (*19*) for goblet, paneth, tuft and enteroendocrine cells, plus proximal and distal enterocytes in aggregated cells from WT, *LApc* and *LApcL* intestines. Wnt/β-catenin signature showing Wnt-high cells, *Lgr5* showing the rapidly proliferating stem cells and *Mki67* showing the proliferating cells.

(**B**) Subclustering of the Wnt/TA cells and quantification of the adenoma cells of the WT, *LApc* and *LApcL* intestinal cells.

(**C**) *Mki67* expression based on scRNA-seq analysis of the *LApc* and *LApcL* adenoma cluster.

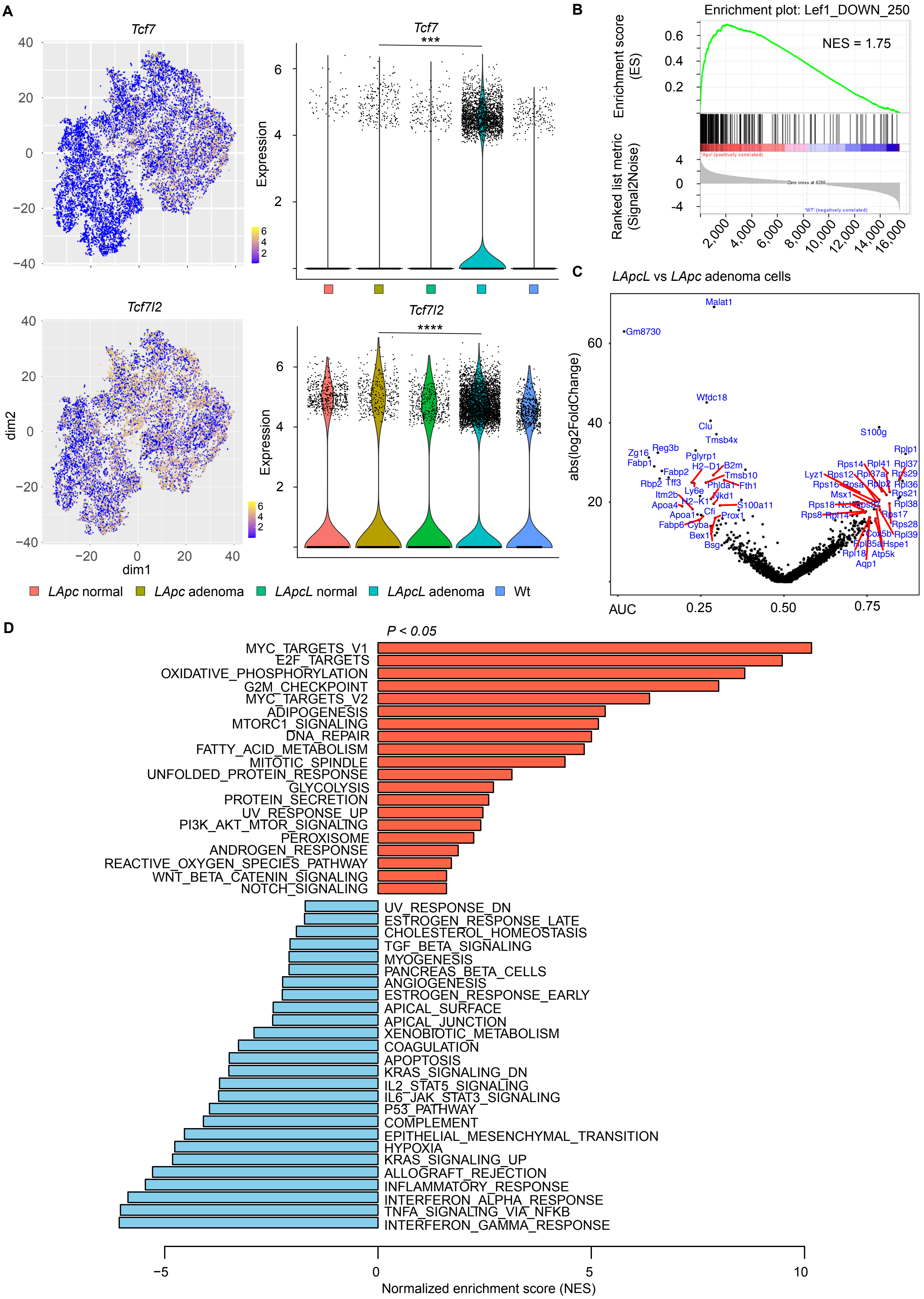

A
Tcf7
Tcf7l2
dim1
dim2
Expression
***
****
LApc normal
LApc adenoma
LApcL normal
LApcL adenoma
Wt
B
Enrichment plot: Lef1_DOWN_250
NES = 1.75
Enrichment score (ES)
Ranked list metric (Signal2Noise)
Zero cross at 8289
2,000
4,000
6,000
8,000
10,000
12,000
14,000
16,000
C
LApcL vs LApc adenoma cells
abs(log2FoldChange)
AUC
Malat1
Gm8730
Wfdc18
Clu
Tmsb4x
S100g
Rplp1
Zg16
Reg3b
Fabp1
Pglyrp1
H2-D1
B2m
Fabp2
Tmsb10
Rbp2
Tff3
Phlda1
Fth1
Ly6e
Itm2b
H2-K1
Nkd1
Apoa4
S100a11
Apoa1
Cfi
Prox1
Fabp6
Cyba
Bex1
Bsg
Rps14
Rpl41
Rpl37
Lyz1
Rps12
Rpl37a
Rps29
Rps16
Rpsa
Rplp2
Rpl36
Msx1
Rps21
Rps18
Rpl38
Rps8
Rpl14
Rps17
Rps28
Cox5b
Rpl39
Rpl35a
Hspe1
Rpl18
Atp5k
Aqp1
D
P < 0.05
MYC_TARGETS_V1
E2F_TARGETS
OXIDATIVE_PHOSPHORYLATION
G2M_CHECKPOINT
MYC_TARGETS_V2
ADIPOGENESIS
MTORC1_SIGNALING
DNA_REPAIR
FATTY_ACID_METABOLISM
MITOTIC_SPINDLE
UNFOLDED_PROTEIN_RESPONSE
GLYCOLYSIS
PROTEIN_SECRETION
UV_RESPONSE_UP
PI3K_AKT_MTOR_SIGNALING
PEROXISOME
ANDROGEN_RESPONSE
REACTIVE_OXYGEN_SPECIES_PATHWAY
WNT_BETA_CATENIN_SIGNALING
NOTCH_SIGNALING
UV_RESPONSE_DN
ESTROGEN_RESPONSE_LATE
CHOLESTEROL_HOMEOSTASIS
TGF_BETA_SIGNALING
MYOGENESIS
PANCREAS_BETA_CELLS
ANGIOGENESIS
ESTROGEN_RESPONSE_EARLY
APICAL_SURFACE
APICAL_JUNCTION
XENOBIOTIC_METABOLISM
COAGULATION
APOPTOSIS
KRAS_SIGNALING_DN
IL2_STAT5_SIGNALING
IL6_JAK_STAT3_SIGNALING
P53_PATHWAY
COMPLEMENT
EPITHELIAL_MESENCHYMAL_TRANSITION
HYPOXIA
KRAS_SIGNALING_UP
ALLOGRAFT_REJECTION
INFLAMMATORY_RESPONSE
INTERFERON_ALPHA_RESPONSE
TNFA_SIGNALING_VIA_NFKB
INTERFERON_GAMMA_RESPONSE
Normalized enrichment score (NES)

Fig. S4. scRNAseq analysis of *LApc* and *LApcL* adenoma cells

(**A**) *Tcf7* and *Tcf7l2* expression analysis based on scRNA-seq analysis of the WT, *LApc* and *LApcL* Wnt/TA clusters. P***<0.005, P****<0.001.

(**B**) Enrichment plot showing comparison of the *Apc* signature versus transcriptional signatures of the 250 most downregulated genes of *LApcL* vs *LApc* adenoma cells.

(**C**) Volcano plot of differentially expressed genes in the *LApcL* vs *LApc* adenoma cluster.

(**D**) GSEA analysis showing enriched Hallmark pathways based on MSigDB analysis of the *LApcL* vs *LApc* adenoma clusters. P<0.05. Note, that interestingly several pro-inflammatory markers were downregulated by *Lef1* deletion.

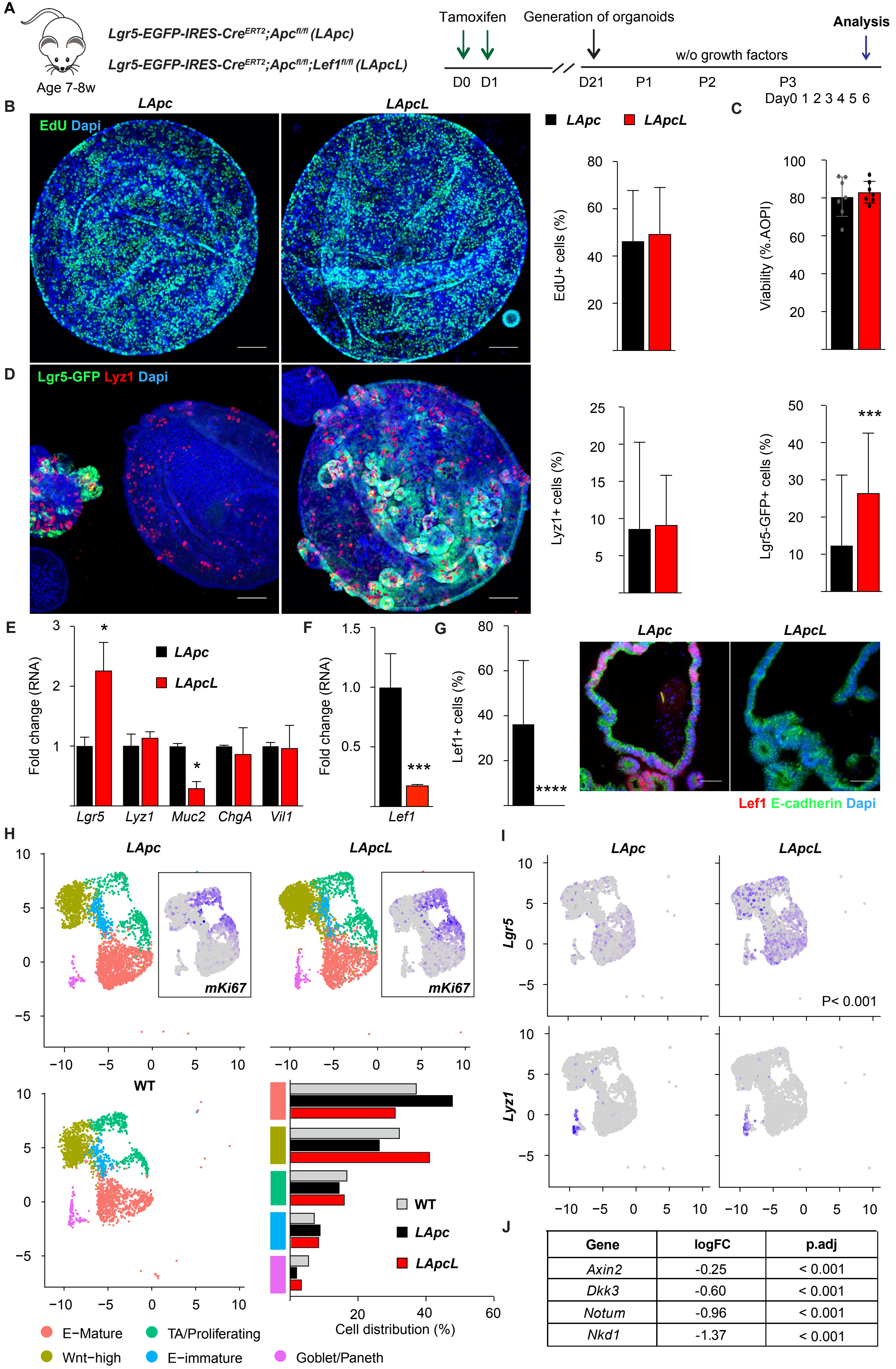

Gene	logFC	p.adj
Axin2	-0.25	< 0.001
Dkk3	-0.60	< 0.001
Notum	-0.96	< 0.001
Nkd1	-1.37	< 0.001

Fig. S5. ***Lef1*** **deletion leads to increased Lgr5 expression in** ***LApcL*** **organoid cultures**

(**A**) Schematic of the experiment. *LApc* and *LApcL* mice treated with two doses of tamoxifen at the age of 7-8 weeks, followed by intestinal crypt isolation 21 days thereafter. The organoids were cultured without growth factors and analyzed six days after the third subculture.

(**B**) Immunostaining and quantification (mean±SD) of the percentage of EdU+ cells. Magnification 10x. Scale bars 100 μm.

(**C**) Cell viability (%). AOPI = Acridine orange/propidium iodine.

(**D**) Immunostaining and quantification (mean±SD) of the percentage of Lgr5-GFP+ and Lyz1+ cells. Magnification 10x. Scale bar 100 μm, ****$P<0.001$.

(**E**) QPCR analysis of *Lgr5*, *Lyz1*, *Muc2*, *ChgA* and *Vil1* expression. *$P<0.05$.

(**F**) QPCR Analysis of *Lef1* expression. ***$P<0.005$.

(**G**) Lef1 and E-cadherin immunostaining and quantification (mean±SD) of the percentage of Lef1 positive cells among approximately 1,500 cells. Scale bar 100 μm, ****$P<0.001$

(**H**) UMAP visualization of scRNA-seq results, *mKi67* transcript and cell distribution percentages in the indicated scRNA-seq clusters.

(**I**) *Lgr5* and *Lyz1* expression in the scRNA-seq clusters.

(**J**) Fold difference of the Wnt antagonist transcripts *Axin2*, *Dkk3*, *Notum* and *Nkd1* in the Wnt-high clusters of *LApcL* vs *LApc* organoids.

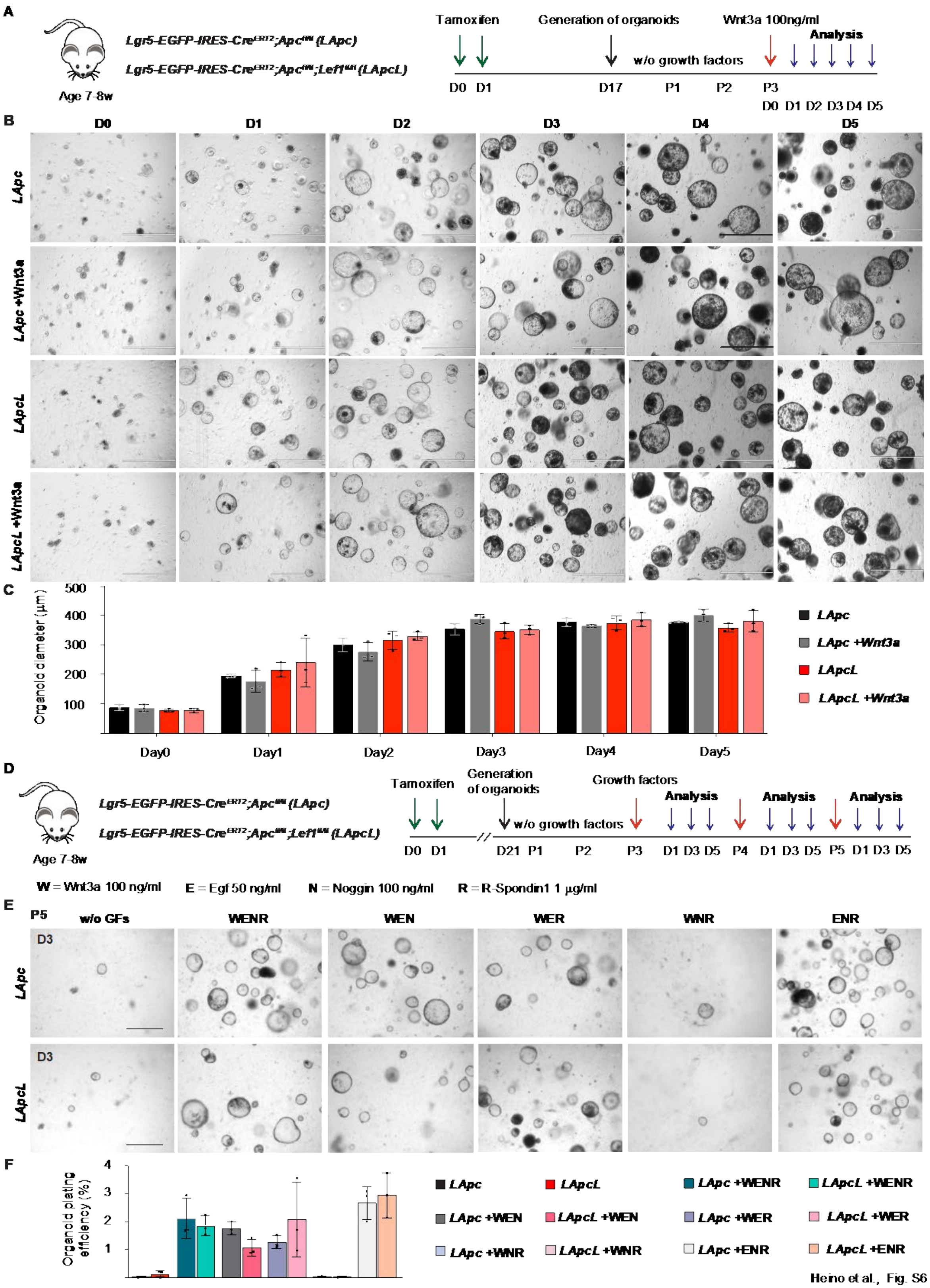

Heino et al., Fig. S6

Fig. S6. Growth and plating efficiency of *LApc* and *LApcL* organoids treated with the indicated factors.

(**A**) Intestinal crypts were isolated 17 days after tamoxifen treatment and the organoids were cultured without growth factors for two passages. Wnt3a was added to the culture medium after the third subculture and the organoid growth was analyzed daily for five days.

(**B**) Images of the *LApc* and *LApcL* organoids. Scale bar 1000 μm.

(**C**) Quantification of organoid diameters.

(**D**) Intestinal crypts were isolated 21 days after tamoxifen treatment, cultured without growth factors for two passages. Upon the third passage, Wnt3a (W), Egf (E), Noggin (N) and/or R-Spondin1 (R) were added to the culture medium as indicated, and organoid growth was analyzed for three passages.

(**E**) Images of the *LApc* and *LApcL* organoids three days after the fifth passage. Scale bars: 1000 μm.

(**F**) Quantification of the new organoid formation capacity (%) of the cells in *LApc* and *LApcL* organoids upon passage five.

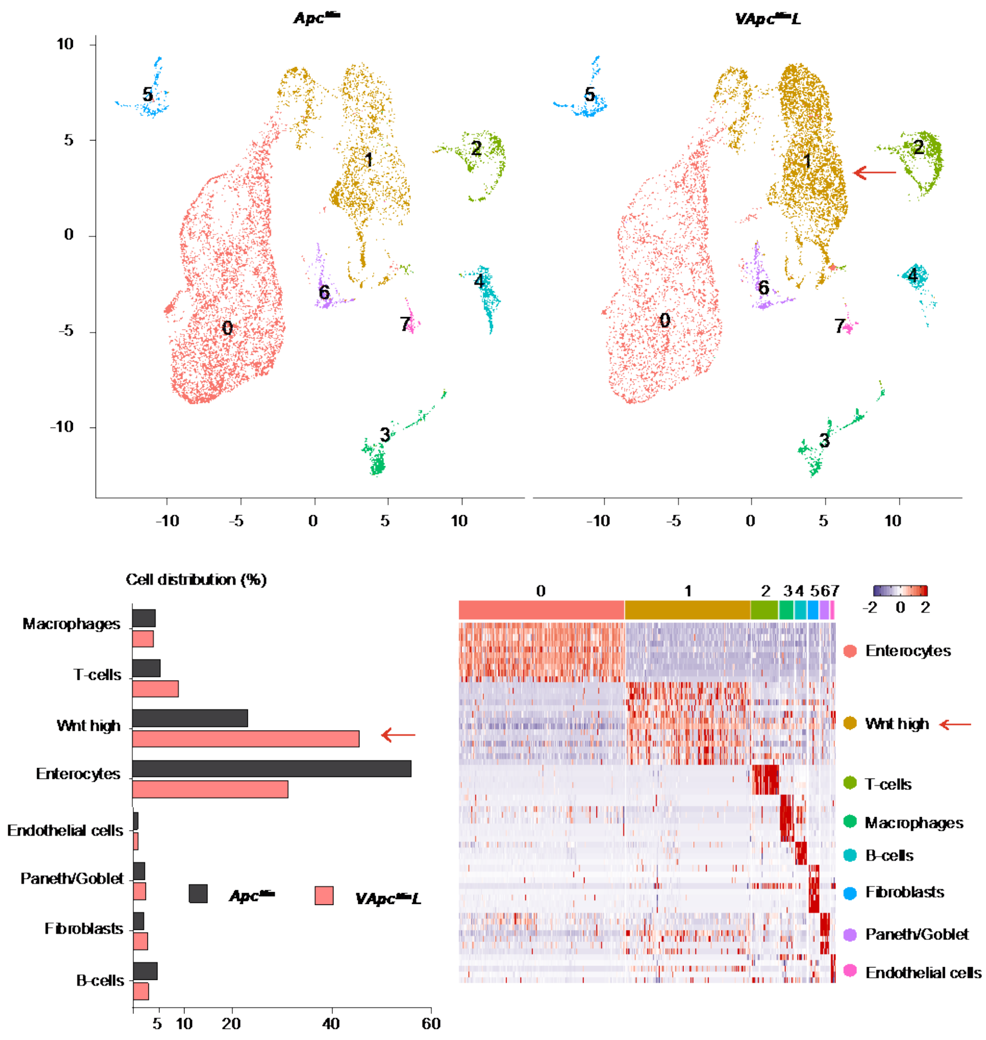
Apc^Min
VApc^Min L
Cell distribution (%)
Macrophages
T-cells
Wnt high
Enterocytes
Endothelial cells
Paneth/Goblet
Fibroblasts
B-cells
T-cells
Macrophages
B-cells
Fibroblasts
Paneth/Goblet
Endothelial cells

Fig. S7. Identification of UMAP clusters and their proportions in *Apc^Min^* and *VApc^Min^L* tumors

Definition and percentages of cells in each cluster of the UMAP clusters of the Apc^{Min} and $VApc^{Min}L$ tumor cells. Arrows points to the Wnt-high cluster.

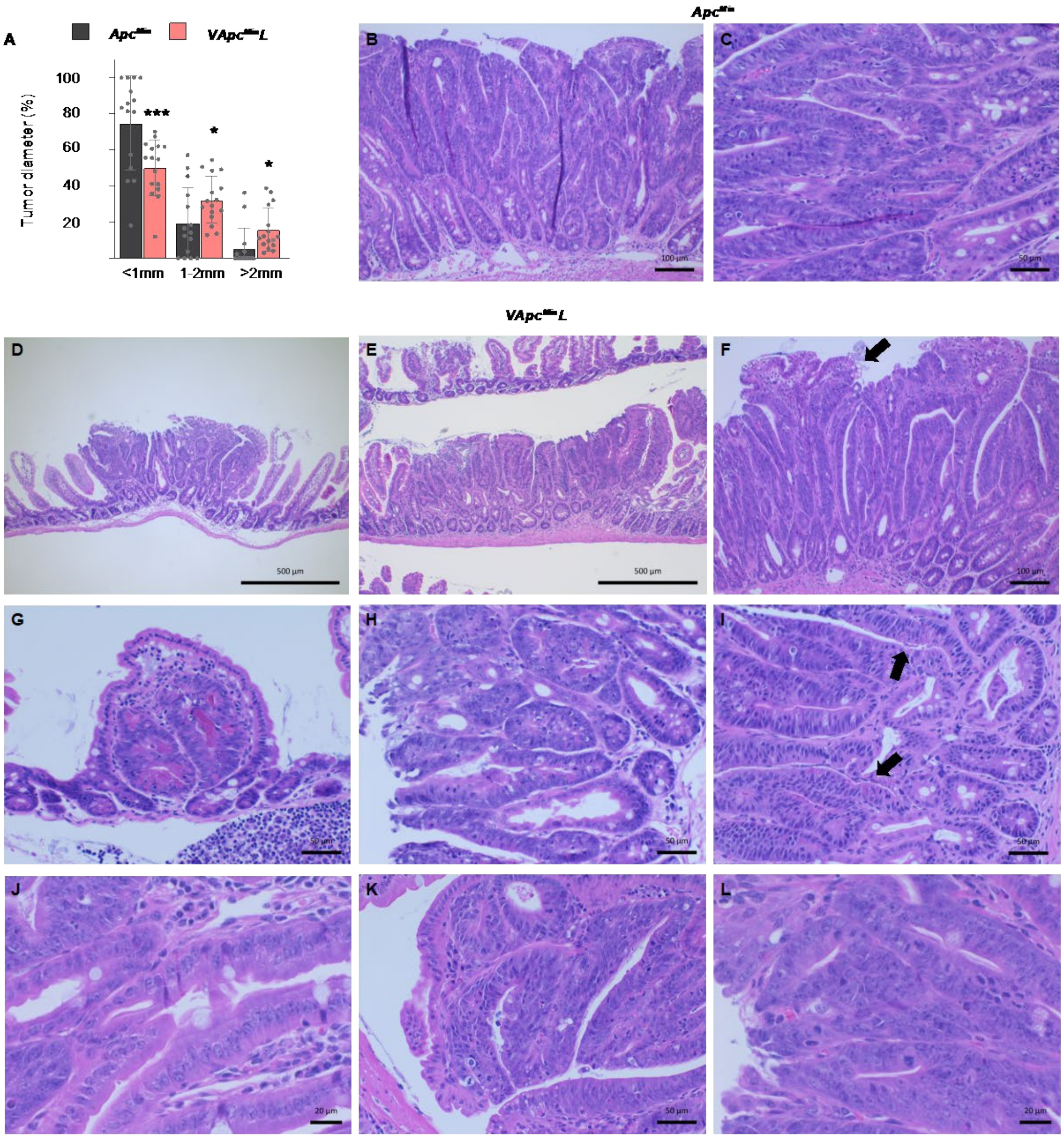

Heino et al., Fig. S8

Fig. S8. Histological analysis of Apc^{Min} and $VApc^{Min}L$ tumors

(**A**) Quantification of the diameter of *Apc^{Min}* and *VApc^{Min}L* small intestinal tumors at 11 weeks of age. *Apc^{Min}* (n=15), *VApc^{Min}L* (n=16), $P^{*}<0.05$, $P^{***}<0.005$.

(**B-L**) Hematoxylin-Eosin stainings of *Apc^{Min}* and *VApc^{Min}L* small intestinal tumors at 11 weeks of age. (**B and C**) sessile adenoma, high grade. Vertically uniform cell type. (**B**) Magnification 10x, scale bar 100 µm, (**C**) 20x, Scale bar 50 µm. (**D**) *VApc^{Min}L* broad-based tubulovillous adenoma, high grade. 5x, scale bar 500 µm. (**E**) *VApc^{Min}L* sessile tubulovillous adenoma, high-grade. 5x, scale bar 500 µm. (**F**) *VApc^{Min}L* sessile adenoma. Villous structure with unaffected surface epithelium (arrow). 10x, scale bar 100 µm. (**G**). *VApc^{Min}L* atypical hyperplastic focus. 20x,scale bar 50 µm. (**H**) *VApc^{Min}L* layered pattern in sessile high-grade adenoma. Note the thin mucosa. 20x, scale bar 50 µm. (**I**) *VApc^{Min}L* layered pattern, basal-mid areas. Arrows point to Paneth cells. 20x, scale bar 50 µm. (**J**) *VApc^{Min}L* cellular and nuclear features in mid area. 40x, scale bar 20 µm. (**K**) *VApc^{Min}L* superficial dysplastic area. 20x, scale bar 50 µm. (**L**) *VApc^{Min}L* cellular and nuclear features in a dysplastic area. 40x, scale bar 20 µm.

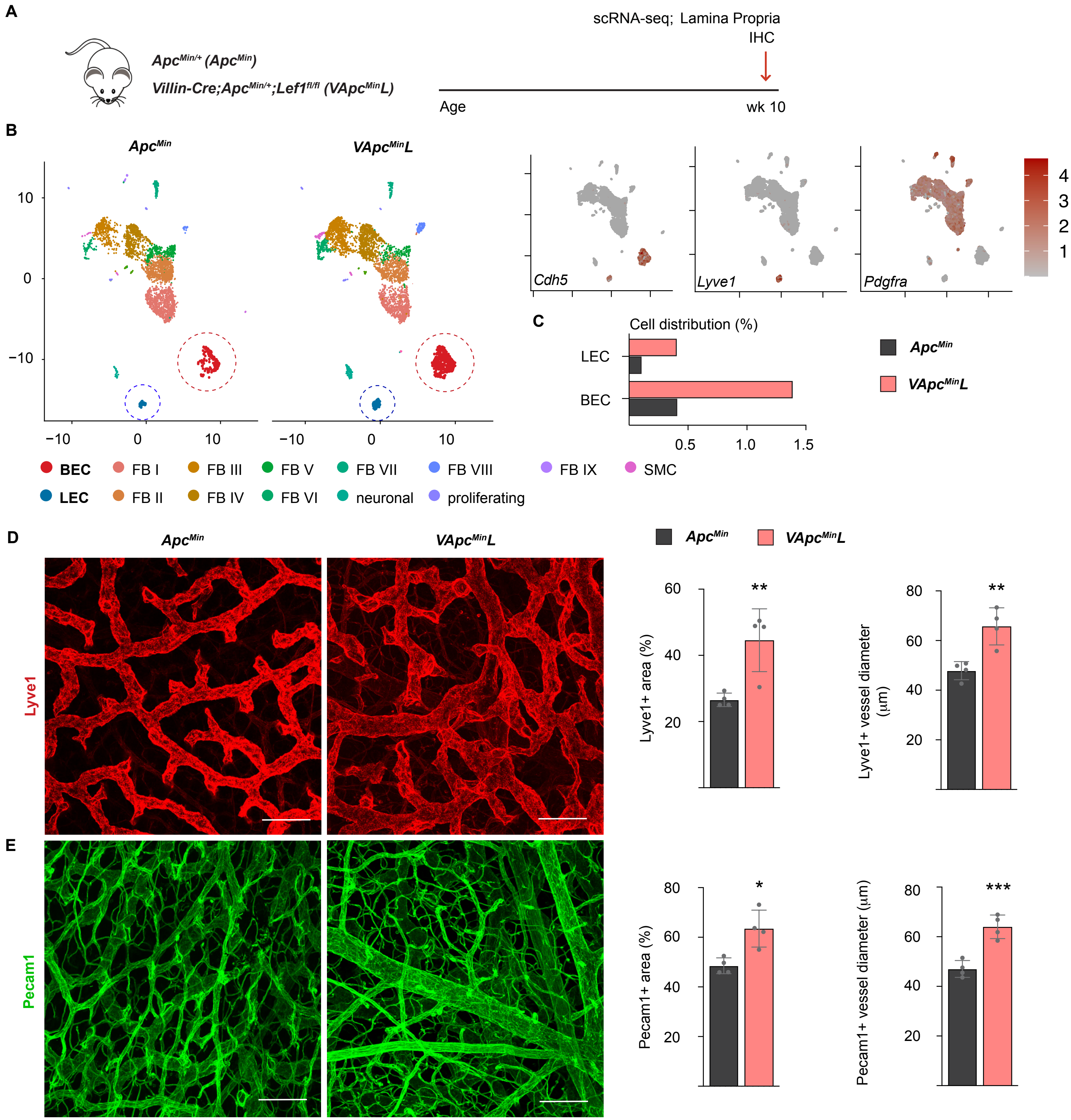

A
scRNA-seq; Lamina Propria
IHC
ApcMin/+ (ApcMin)
Villin-Cre;ApcMin/+;Lef1fl/fl (VApcMinL)
Age
wk 10
B
ApcMin
VApcMinL
Cdh5
Lyve1
Pdgfra
C
Cell distribution (%)
LEC
BEC
ApcMin
VApcMinL
BEC FB I FB III FB V FB VII FB VIII FB IX SMC
LEC FB II FB IV FB VI neuronal proliferating
D
ApcMin
VApcMinL
Lyve1
Lyve1+ area (%)
Lyve1+ vessel diameter (μm)
E
Pecam1
Pecam1+ area (%)
Pecam1+ vessel diameter (μm)

Fig. S9. *Lef1* deletion increases vessel area and diameter in the *VApc^Min L* intestine.

(**A**) Schematic of the experiment. The *Apc^Min* and *VApc^Min L* mice were analyzed at the age of 10 weeks.

(**B**) Definition of each cluster of the UMAP of the *Apc^Min* and *VApc^Min L* lamina propria.

(**C**) Percentages of LEC and BEC clusters of the *Apc^Min* and *VApc^Min L* lamina propria.

(**D-E**) Immunofluorescent staining and quantification of (**D**) Lyve1+ and (**E**) Pecam1+ vessel area and diameter in *Apc^Min* and *VApc^Min L* intestine. Data are shown as mean±SD. Scale bars 50 µm, n=4 per group, P*<0.05, P**<0.01, P***<0.005.

Table S1. scRNA-seq analysis of differentially expressed genes from *LApcL* vs *LApc* adenoma cells.

Table S2. Functional gene set enrichment analysis (FGSEA) of the biological processes of the gene ontology (GOBP) from *LApcL* vs *LApc* adenoma cells.
