# Supplementary material for: Lef1 restricts ectopic crypt formation and tumor cell growth in intestinal adenomas
Source: Sci Adv. 2021 Nov 17;7(47):eabj0512. doi: 10.1126/sciadv.abj0512 (PMC8598008; doi:10.1126/sciadv.abj0512)
Supplement: Supplementary file 3 — Correction 20 May 2022: Due to a rendering error, the axes for Figures S4(A), S5(H) and S9(B) were illegible. The supplementary materials PDF has now been corrected. The original version is available here: [file sciadv.abj0512_sm.v1.pdf]

Science Advances
AAAS

Supplementary Materials for

Lef1 restricts ectopic crypt formation and tumor cell growth in intestinal adenomas

Sarika Heino, Shentong Fang, Marianne Lähde, Jenny Högström, Sina Nassiri, Andrew Campbell, Dustin Flanagan, Alexander Raven, Michael Hodder, Nadia Nasreddin, Hai-Hui Xue, Mauro Delorenzi, Simon Leedham, Tatiana V. Petrova, Owen Sansom, Kari Alitalo*

*Corresponding author. Email: kari.alitalo@helsinki.fi

Published 17 November 2021, *Sci. Adv.* **7**, eabj0512 (2021)
DOI: 10.1126/sciadv.abj0512

The PDF file includes:

Figs. S1 to S9
Legends for tables S1 and S2

Other Supplementary Material for this manuscript includes the following:

Tables S1 and S2

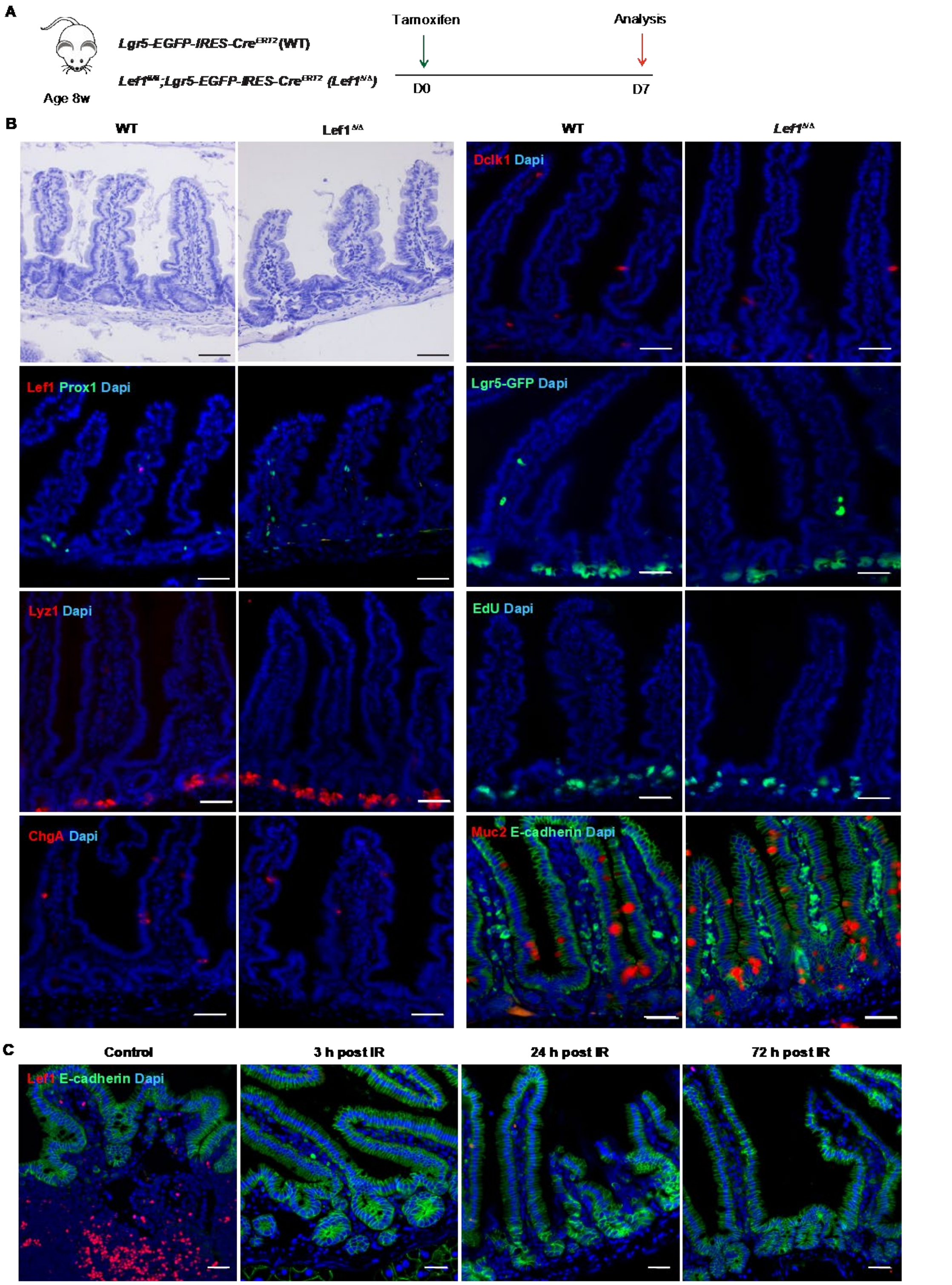

Heino et al., Fig S1

Figure S1. Lef1 is not required for homeostasis or regeneration of the healthy intestine.

(**A**) Lgr5-EGFP-IRES-Cre^{ERT2} (WT) and Lgr5-EGFP-IRES-Cre^{ERT2};Lef1$^{fl/fl}$ (Lef1$^{\Delta/\Delta}$) mice received a single dose of tamoxifen at the age of 8 weeks and were analyzed one week thereafter.

(**B**) Hemalum staining and staining for Lef1, Prox1, Lysozyme1 (Lyz1), Chromogranin A (ChgA), Doublecortin like kinase 1 (Dclk1), Lgr5-GFP, EdU, Mucin2 (Muc2) and E-cadherin in WT and Lef1$^{\Delta/\Delta}$ intestine. Scale bars 50µm, n= 3 mice per group.

(**C**) Lef1 and E-cadherin staining in WT intestine at 3, 24 and 72 hours after 10 Gy irradiation. Scale bars 20 µm, n= 3 mice per time point. Lef1 expression in the T-cells of Peyer's patches was used as a positive control for Lef1 staining.

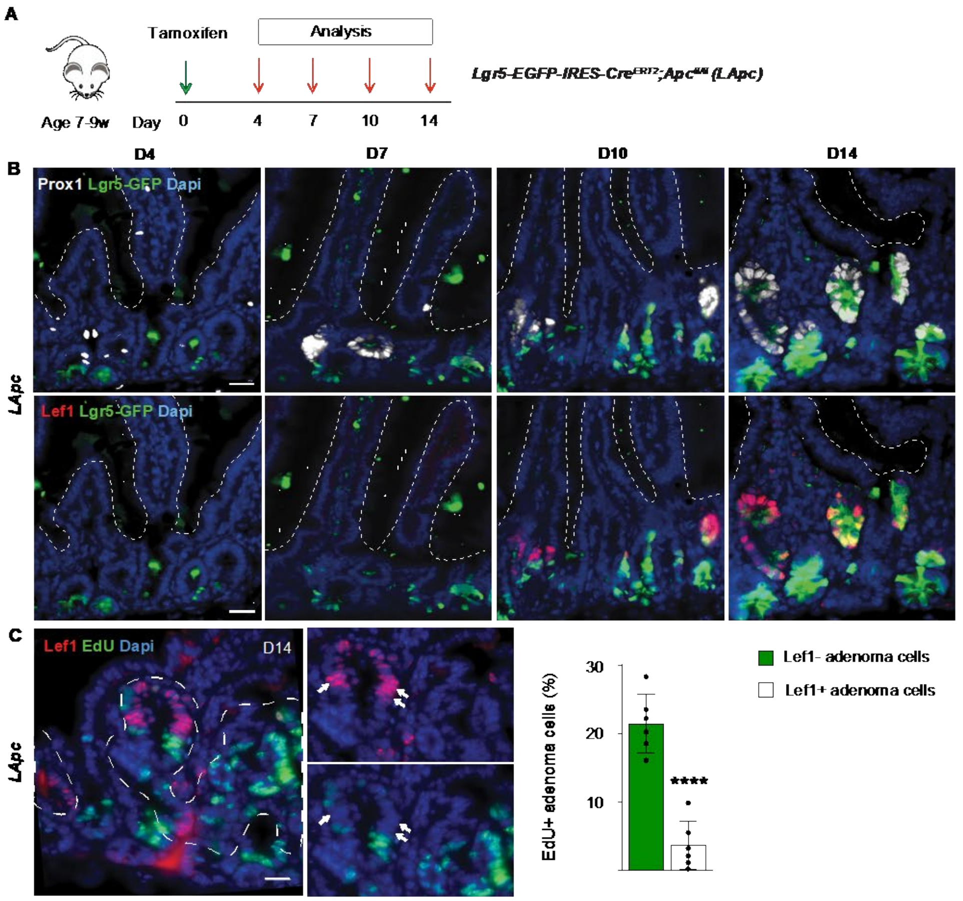

Heino et al., Fig. S2

Figure S2. Lef1 is induced in Lgr5+;Prox1+ cells after *Apc* deletion.

(A) Schematic of the experiment. *Lgr5-EGFP-IRES-Cre*^{ERT2}*;Apc*$^{fl/fll}$ *(LApc)* mice received a single dose of tamoxifen at the age of 7-9 weeks and were analyzed at the time points indicated.

(B) Immunofluorescent staining of Prox1, Lef1 and Lgr5-GFP 4, 7, 10 and 14 days after tamoxifen administration. Dashed line indicates the luminal surface of the intestine. Scale bar 20 μm, n=3 per time point.

(C) EdU and Lef1 staining and quantification 14 days after *Apc* deletion. Dashed line indicates nuclear β-catenin+ area. Arrows point Lef1+EdU- cells. Scale bar 20 μm, n=6, P****<0.001. Data are shown as mean±SD.

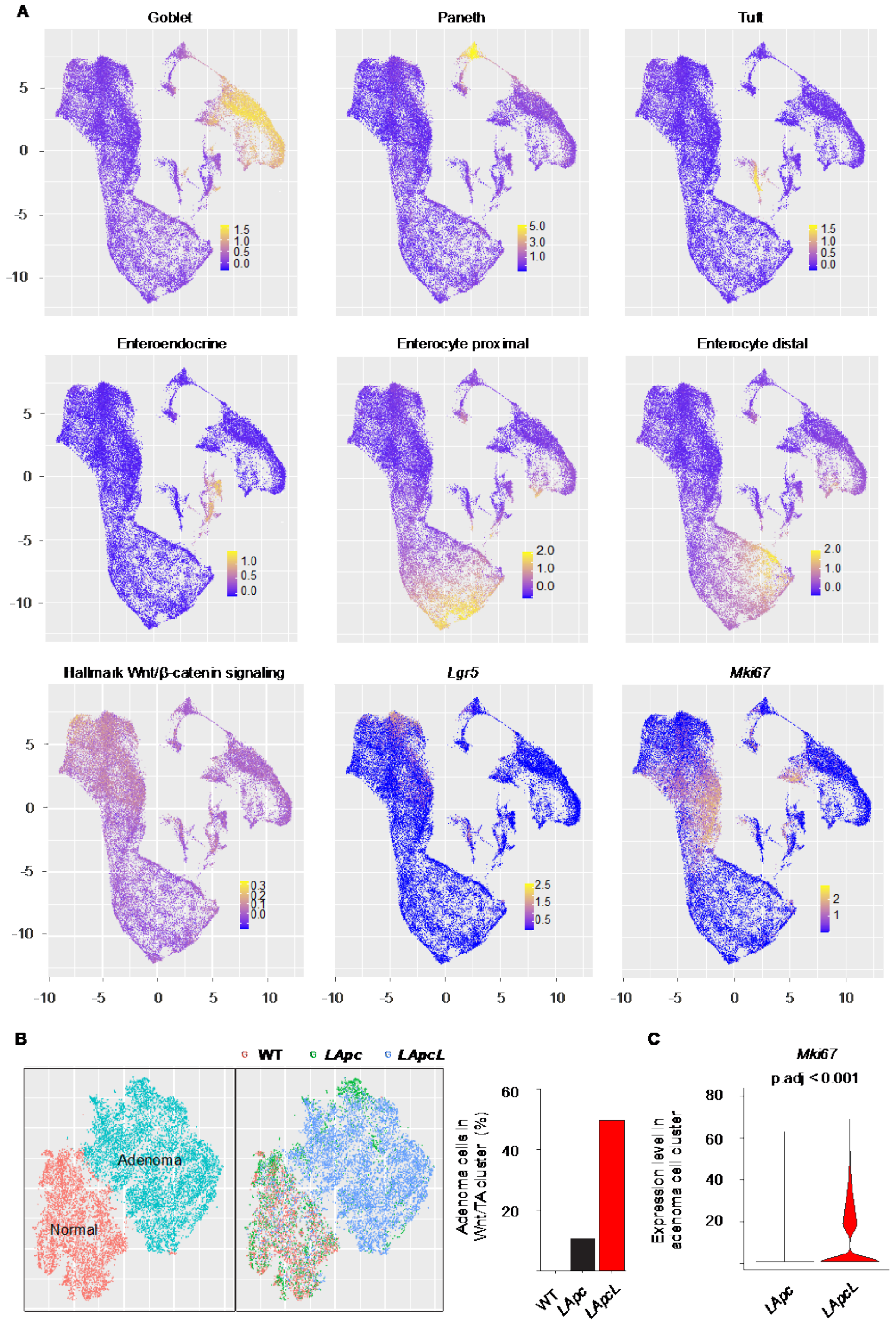

Heino et al., Fig. S3

Fig. S3. Cluster identification among intestinal EpCAM+ single cells

(**A**) Module enrichment scores of signatures (*19*) for goblet, paneth, tuft and enteroendocrine cells, plus proximal and distal enterocytes in aggregated cells from WT, *LApc* and *LApcL* intestines. Wnt/β-catenin signature showing Wnt-high cells, *Lgr5* showing the rapidly proliferating stem cells and *Mki67* showing the proliferating cells.

(**B**) Subclustering of the Wnt/TA cells and quantification of the adenoma cells of the WT, *LApc* and *LApcL* intestinal cells.

(**C**) *Mki67* expression based on scRNA-seq analysis of the *LApc* and *LApcL* adenoma cluster.

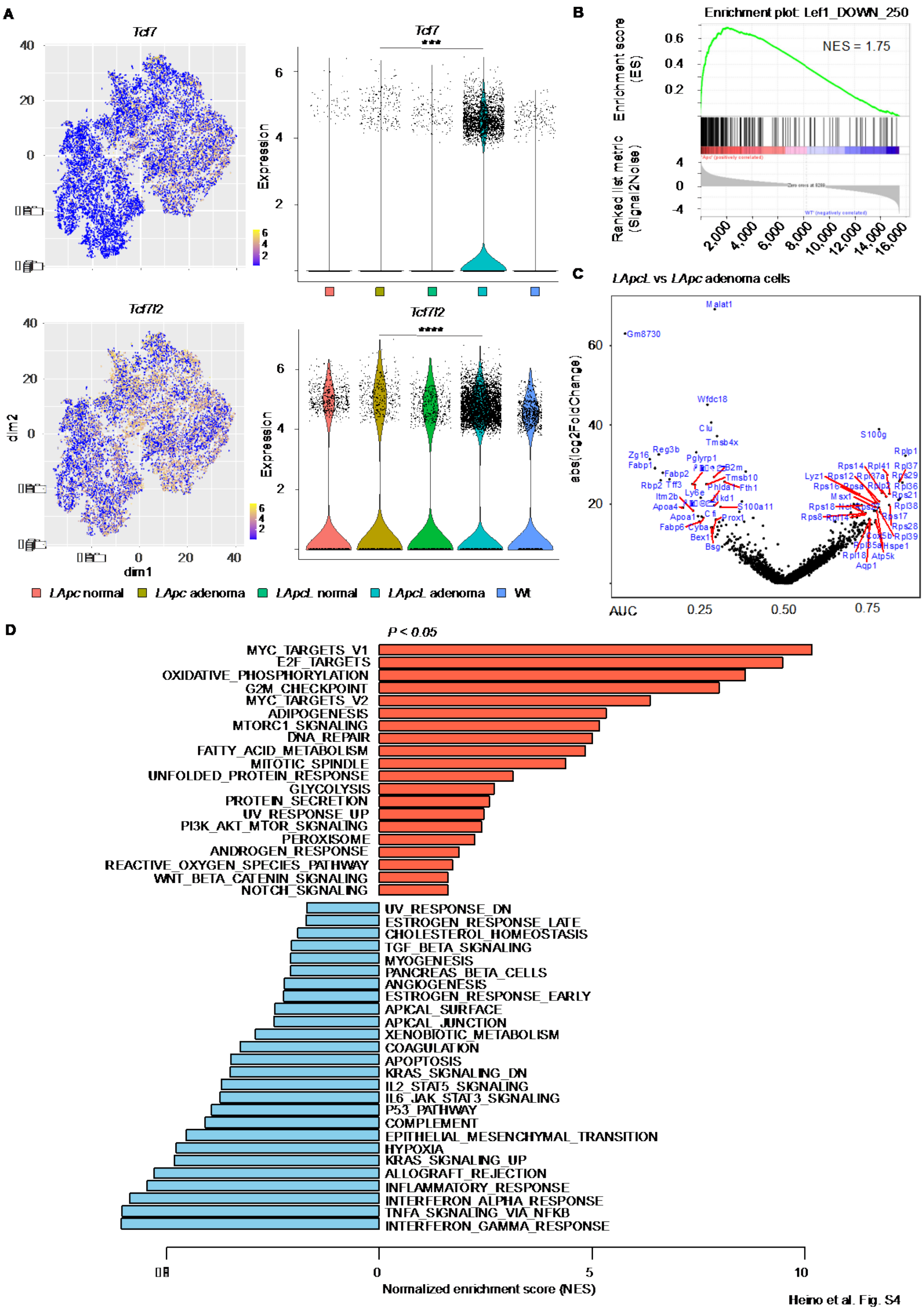

Heino et al. Fig. S4

Fig. S4. scRNAseq analysis of *LApc* and *LApcL* adenoma cells

(**A**) *Tcf7* and *Tcf7l2* expression analysis based on scRNA-seq analysis of the WT, *LApc* and *LApcL* Wnt/TA clusters. P***<0.005, P****<0.001.

(**B**) Enrichment plot showing comparison of the *Apc* signature versus transcriptional signatures of the 250 most downregulated genes of *LApcL* vs *LApc* adenoma cells.

(**C**) Volcano plot of differentially expressed genes in the *LApcL* vs *LApc* adenoma cluster.

(**D**) GSEA analysis showing enriched Hallmark pathways based on MSigDB analysis of the *LApcL* vs *LApc* adenoma clusters. P<0.05. Note, that interestingly several pro-inflammatory markers were downregulated by *Lef1* deletion.

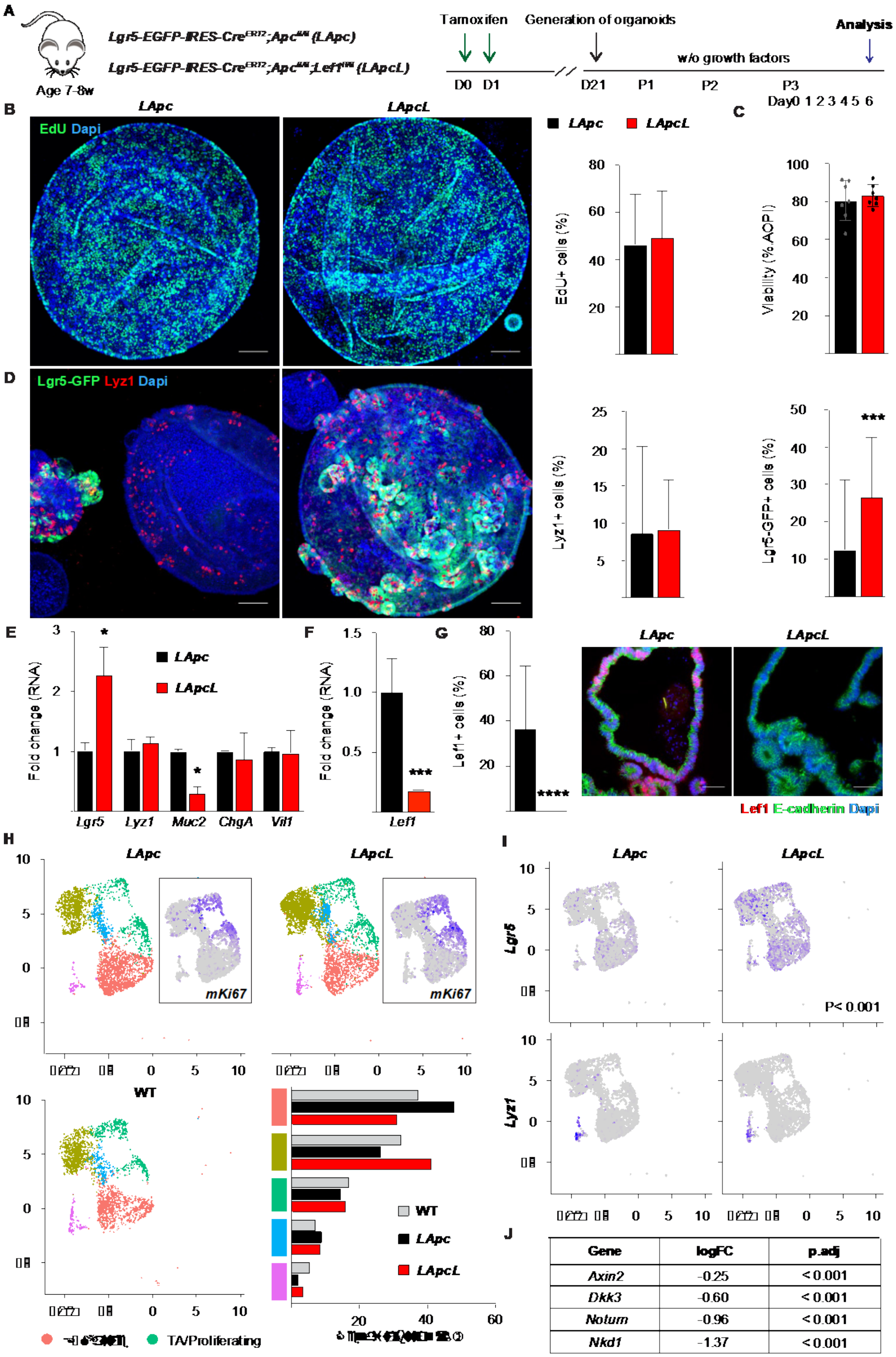

Gene	logFC	p.adj
Axin2	-0.25	< 0.001
Dkk3	-0.60	< 0.001
Notum	-0.96	< 0.001
Nkd1	-1.37	< 0.001

Heino et al., Fig. S5

Fig. S5. ***Lef1* deletion leads to increased Lgr5 expression in *LApcL* organoid cultures**

(**A**) Schematic of the experiment. *LApc* and *LApcL* mice treated with two doses of tamoxifen at the age of 7-8 weeks, followed by intestinal crypt isolation 21 days thereafter. The organoids were cultured without growth factors and analyzed six days after the third subculture.

(**B**) Immunostaining and quantification (mean±SD) of the percentage of EdU+ cells. Magnification 10x. Scale bars 100 μm.

(**C**) Cell viability (%). AOPI = Acridine orange/propidium iodine.

(**D**) Immunostaining and quantification (mean±SD) of the percentage of Lgr5-GFP+ and Lyz1+ cells. Magnification 10x. Scale bar 100 μm, ****P<0.001.

(**E**) QPCR analysis of *Lgr5*, *Lyz1*, *Muc2*, *ChgA* and *Vil1* expression. *P<0.05.

(**F**) QPCR Analysis of *Lef1* expression. ***P<0.005.

(**G**) Lef1 and E-cadherin immunostaining and quantification (mean±SD) of the percentage of Lef1 positive cells among approximately 1,500 cells. Scale bar 100 μm, ****P<0.001

(**H**) UMAP visualization of scRNA-seq results, *mKi67* transcript and cell distribution percentages in the indicated scRNA-seq clusters.

(**I**) *Lgr5* and *Lyz1* expression in the scRNA-seq clusters.

(**J**) Fold difference of the Wnt antagonist transcripts *Axin2*, *Dkk3*, *Notum* and *Nkd1* in the Wnt-high clusters of *LApcL* vs *LApc* organoids.

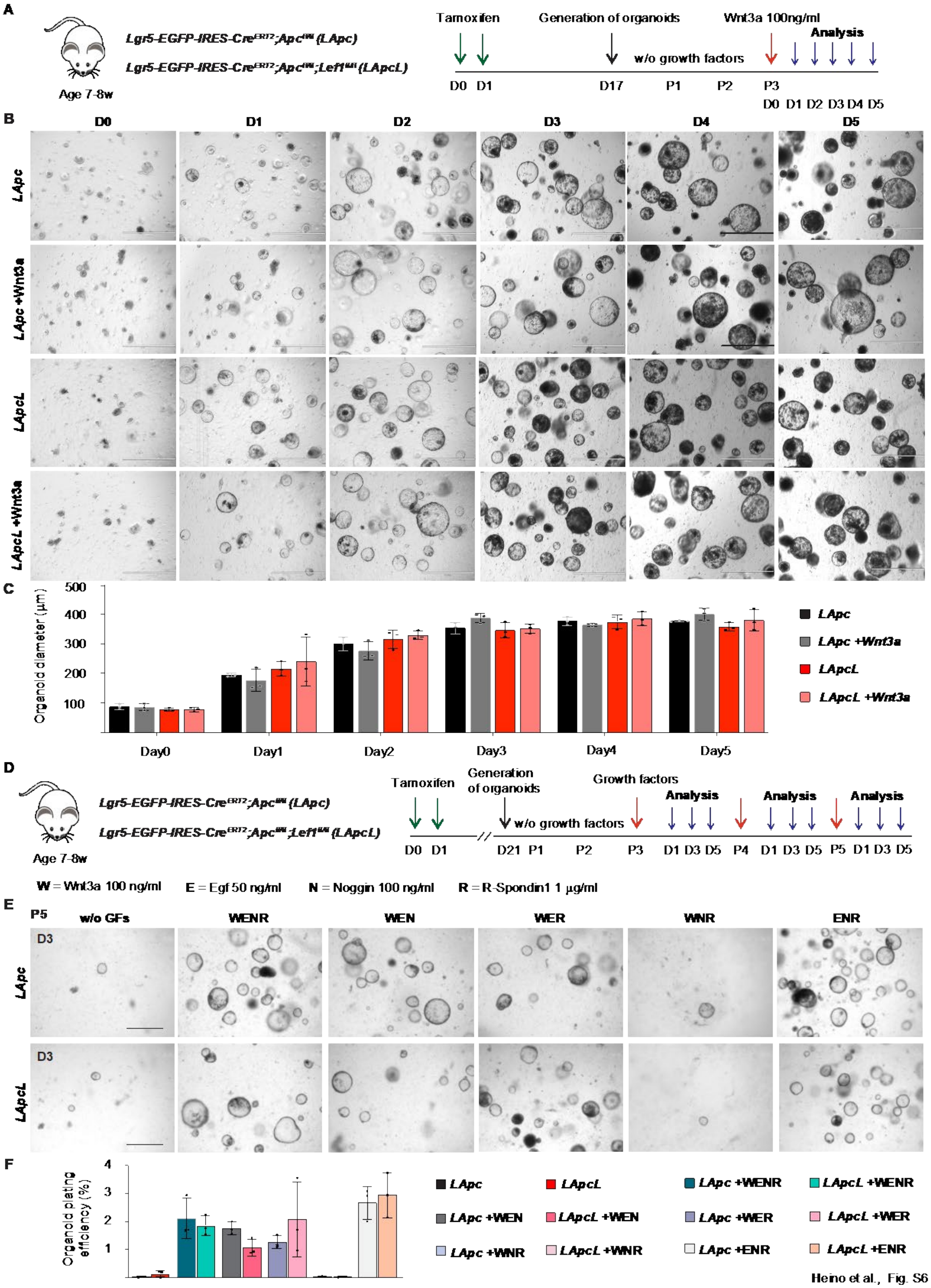

Heino et al., Fig. S6

Fig. S6. Growth and plating efficiency of *LApc* and *LApcL* organoids treated with the indicated factors.

(**A**) Intestinal crypts were isolated 17 days after tamoxifen treatment and the organoids were cultured without growth factors for two passages. Wnt3a was added to the culture medium after the third subculture and the organoid growth was analyzed daily for five days.

(**B**) Images of the *LApc* and *LApcL* organoids. Scale bar 1000 µm.

(**C**) Quantification of organoid diameters.

(**D**) Intestinal crypts were isolated 21 days after tamoxifen treatment, cultured without growth factors for two passages. Upon the third passage, Wnt3a (W), Egf (E), Noggin (N) and/or R-Spondin1 (R) were added to the culture medium as indicated, and organoid growth was analyzed for three passages.

(**E**) Images of the *LApc* and *LApcL* organoids three days after the fifth passage. Scale bars: 1000 µm.

(**F**) Quantification of the new organoid formation capacity (%) of the cells in *LApc* and *LApcL* organoids upon passage five.

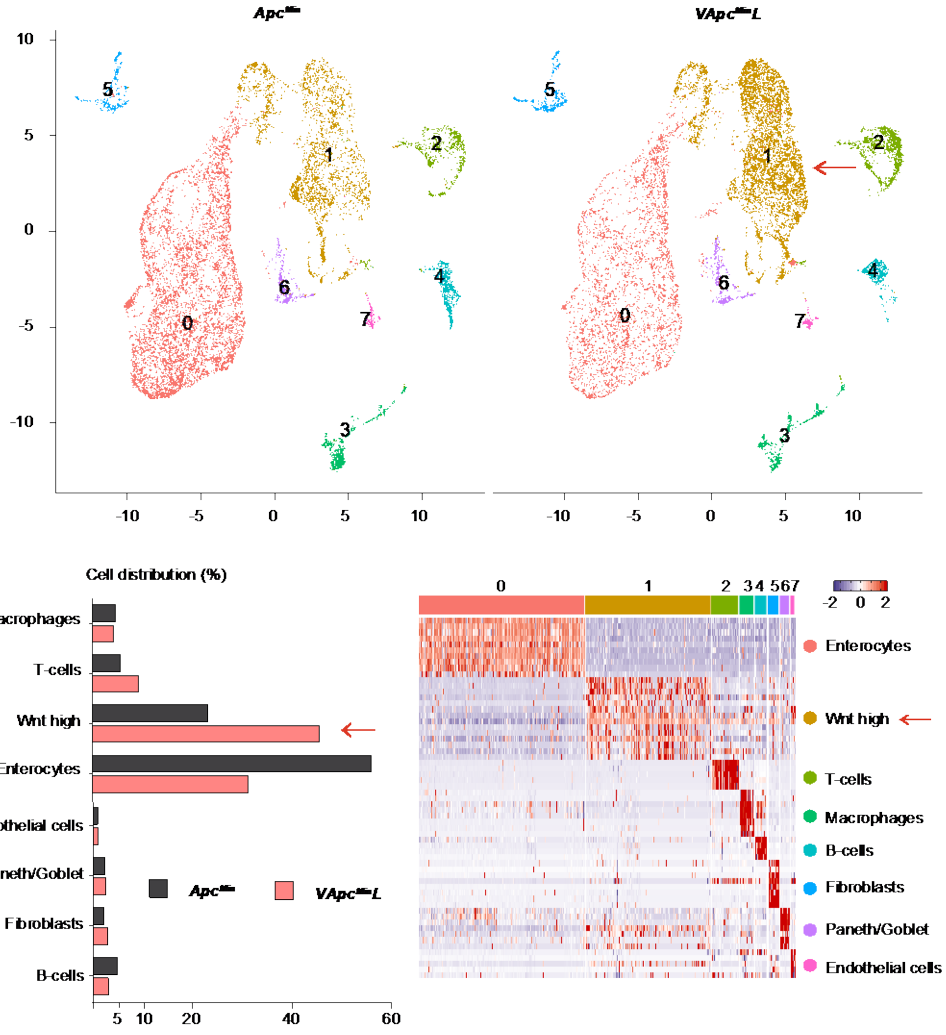

Heino et al., Fig. S7

Fig. S7. Identification of UMAP clusters and their proportions in *Apc*^{Min} and *VApc*^{Min}*L* tumors

Definition and percentages of cells in each cluster of the UMAP clusters of the *Apc*^{Min} and *VApc*^{Min}*L* tumor cells. Arrows points to the Wnt-high cluster.

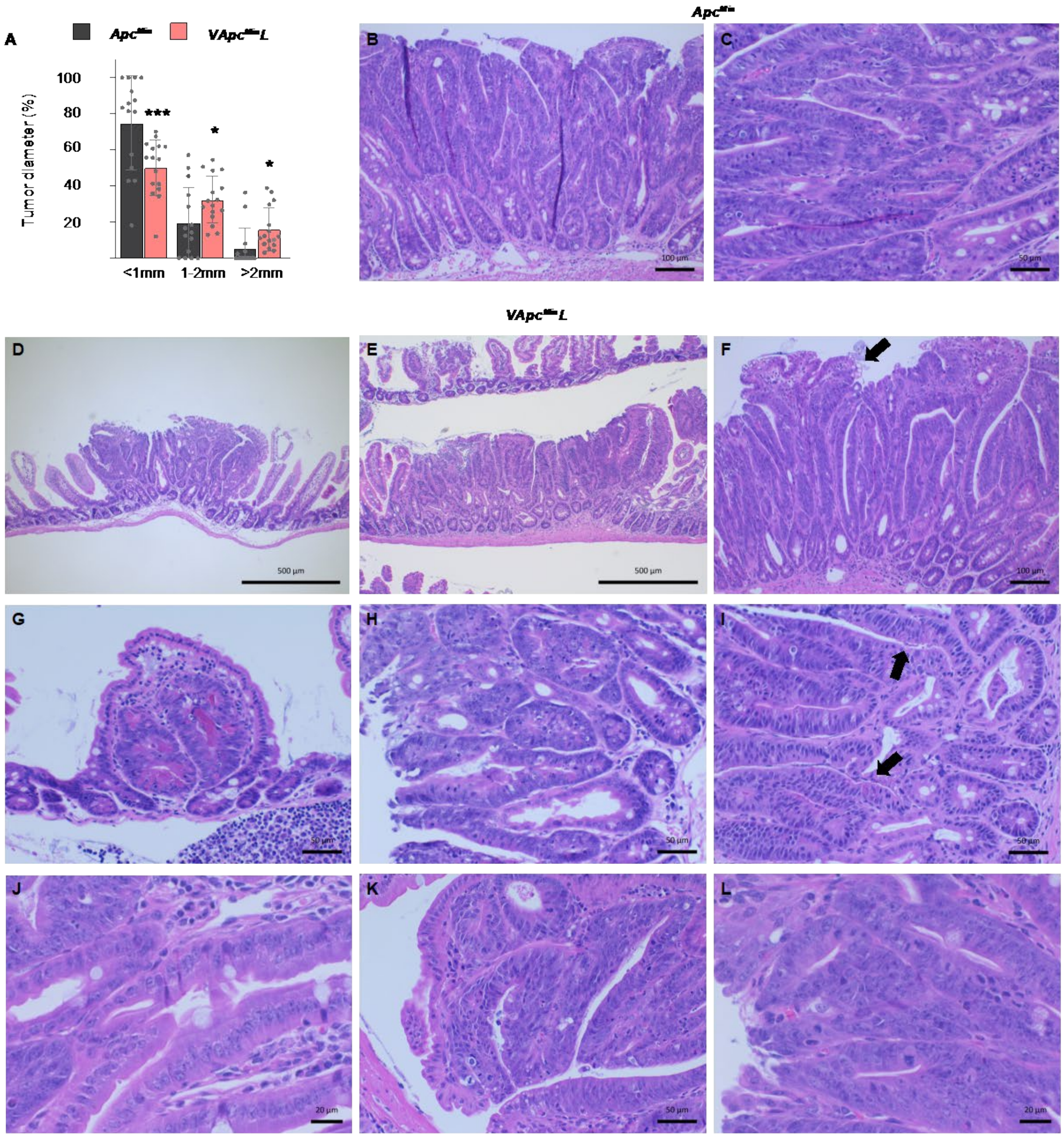

Heino et al., Fig. S8

Fig. S8. Histological analysis of Apc^{Min} and VApc^{Min}L tumors

(**A**) Quantification of the diameter of *Apc^{Min}* and *VApc^{Min}L* small intestinal tumors at 11 weeks of age. *Apc^{Min}* (n=15), *VApc^{Min}L* (n=16), P*<0.05, P***<0.005.

(**B-L**) Hematoxylin-Eosin stainings of *Apc^{Min}* and *VApc^{Min}L* small intestinal tumors at 11 weeks of age. (**B and C**) sessile adenoma, high grade. Vertically uniform cell type. (**B**) Magnification 10x, scale bar 100 µm, (**C**) 20x, Scale bar 50 µm. (**D**) *VApc^{Min}L* broad-based tubulovillous adenoma, high grade. 5x, scale bar 500 µm. (**E**) *VApc^{Min}L* sessile tubulovillous adenoma, high-grade. 5x, scale bar 500 µm. (**F**) *VApc^{Min}L* sessile adenoma. Villous structure with unaffected surface epithelium (arrow). 10x, scale bar 100 µm. (**G**). *VApc^{Min}L* atypical hyperplastic focus. 20x,scale bar 50 µm. (**H**) *VApc^{Min}L* layered pattern in sessile high-grade adenoma. Note the thin mucosa. 20x, scale bar 50 µm. (**I**) *VApc^{Min}L* layered pattern, basal-mid areas. Arrows point to Paneth cells. 20x, scale bar 50 µm. (**J**) *VApc^{Min}L* cellular and nuclear features in mid area. 40x, scale bar 20 µm. (**K**) *VApc^{Min}L* superficial dysplastic area. 20x, scale bar 50 µm. (**L**) *VApc^{Min}L* cellular and nuclear features in a dysplastic area. 40x, scale bar 20 µm.

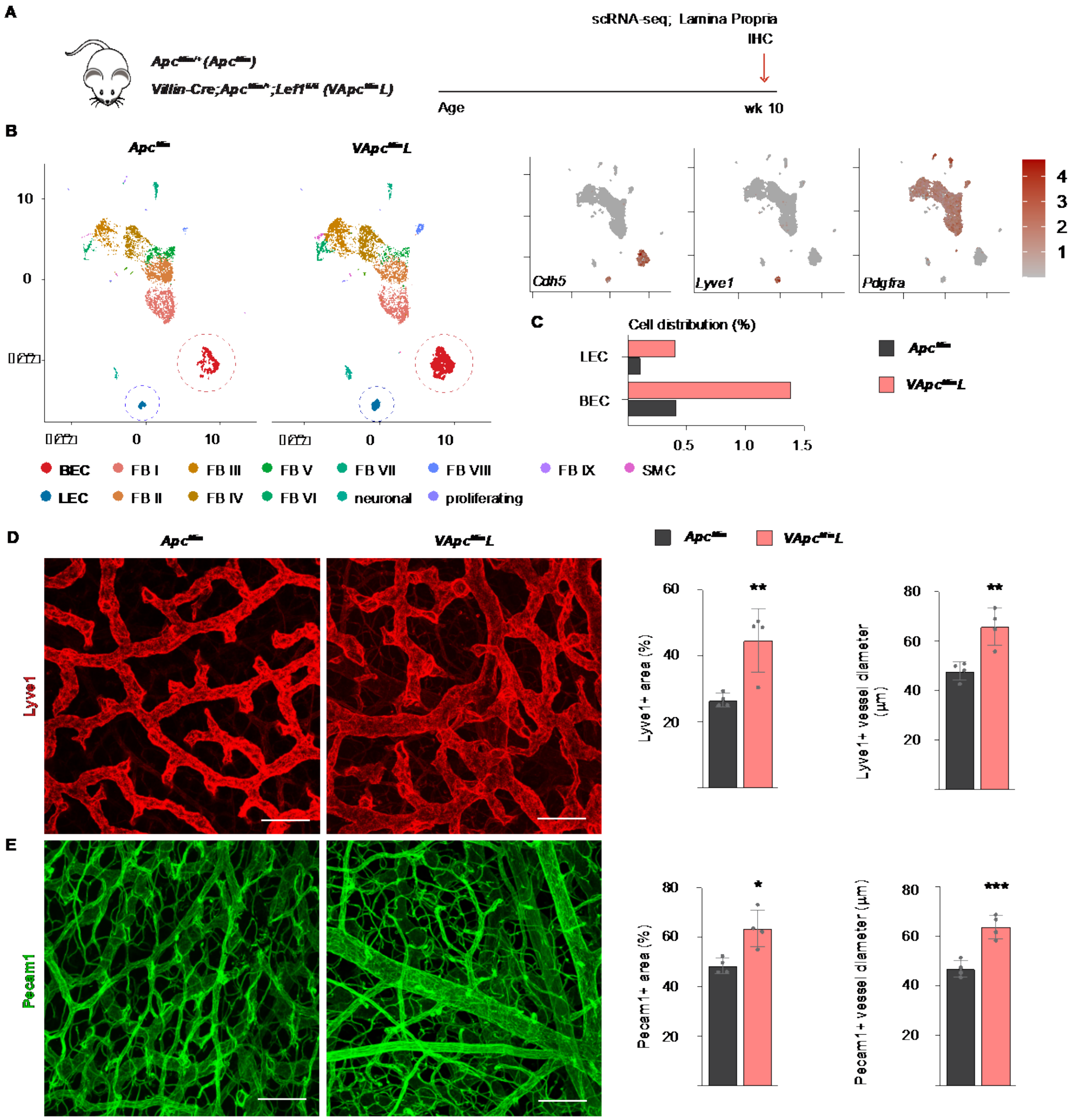

A
scRNA-seq; Lamina Propria
IHC
Age
wk 10
B
Cdh5
Lyve1
Pdgfra
C
Cell distribution (%)
LEC
BEC
BEC
FB I
FB III
FB V
FB VII
FB VIII
FB IX
SMC
LEC
FB II
FB IV
FB VI
neuronal
proliferating
D
Lyve1
Lyve1+ area (%)
Lyve1+ vessel diameter (µm)
E
Pecam1
Pecam1+ area (%)
Pecam1+ vessel diameter (µm)

Fig. S9. *Lef1* deletion increases vessel area and diameter in the *VApc^{Min}L* intestine.

(**A**) Schematic of the experiment. The *Apc^{Min}* and *VApc^{Min}L* mice were analyzed at the age of 10 weeks.

(**B**) Definition of each cluster of the UMAP of the *Apc^{Min}* and *VApc^{Min}L* lamina propria.

(**C**) Percentages of LEC and BEC clusters of the *Apc^{Min}* and *VApc^{Min}L* lamina propria.

(**D-E**) Immunofluorescent staining and quantification of (**D**) Lyve1+ and (**E**) Pecam1+ vessel area and diameter in *Apc^{Min}* and *VApc^{Min}L* intestine. Data are shown as mean±SD. Scale bars 50 µm, n=4 per group, P*<0.05, P**<0.01, P***<0.005.

Table S1. scRNA-seq analysis of differentially expressed genes from *LApcL* vs *LApc* adenoma cells.

Table S2. Functional gene set enrichment analysis (FGSEA) of the biological processes of the gene ontology (GOBP) from *LApcL* vs *LApc* adenoma cells.
